# Detecting, Distinguishing, and Spatiotemporally Tracking Photogenerated Charge and Heat at the Nanoscale

**DOI:** 10.1021/acsnano.3c04607

**Published:** 2023-09-18

**Authors:** Hannah L. Weaver, Cora M. Went, Joeson Wong, Dipti Jasrasaria, Eran Rabani, Harry A. Atwater, Naomi S. Ginsberg

**Affiliations:** † Department of Physics, University of California, Berkeley, California 94720, United States; ‡ Department of Physics, California Institute of Technology, Pasadena, California 91125, United States; ¶ Department of Applied Physics and Materials Science, California Institute of Technology, Pasadena, California 91125, United States; § Department of Chemistry, University of California, Berkeley, California 94720, United States; ∥ Materials Sciences Division, Lawrence Berkeley National Laboratory, Berkeley, California 94720, United States; ⊥ The Sackler Center for Computational Molecular and Materials Science, Tel Aviv University, Tel Aviv, 69978, Israel; # Molecular Biophysics and Integrated Bioimaging Division, Lawrence Berkeley National Laboratory, Berkeley, California 94720, United States; □ Kavli Energy NanoScience Institute, Berkeley, California 94720, United States; △ STROBE NSF Science & Technology Center, Berkeley, California 94720, United States

**Keywords:** excitons, thermometry, energy transport, optical properties, nanoscale

## Abstract

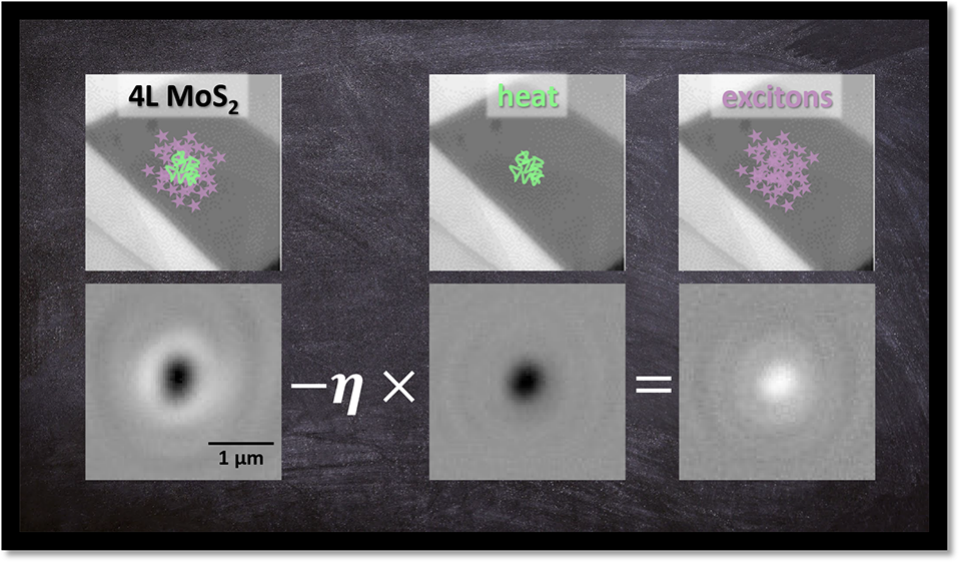

Since dissipative processes are ubiquitous in semiconductors, characterizing how electronic and thermal energy transduce and transport at the nanoscale is vital for understanding and leveraging their fundamental properties. For example, in low-dimensional transition metal dichalcogenides (TMDCs), excess heat generation upon photoexcitation is difficult to avoid since even with modest injected exciton densities exciton–exciton annihilation still occurs. Both heat and photoexcited electronic species imprint transient changes in the optical response of a semiconductor, yet the distinct signatures of each are difficult to disentangle in typical spectra due to overlapping resonances. In response, we employ stroboscopic optical scattering microscopy (stroboSCAT) to simultaneously map both heat and exciton populations in few-layer MoS_2_ on relevant nanometer and picosecond length- and time scales and with 100-mK temperature sensitivity. We discern excitonic contributions to the signal from heat by combining observations close to and far from exciton resonances, characterizing the photoinduced dynamics for each. Our approach is general and can be applied to any electronic material, including thermoelectrics, where heat and electronic observables spatially interplay, and it will enable direct and quantitative discernment of different types of coexisting energy without recourse to complex models or underlying assumptions.

Heat and charge coexist in many semiconducting materials following photoexcitation or electrical injection. Here “heat” refers to heating induced lattice fluctuations, and “charge” refers to excited state electrons, holes, and their correlated combinations, e.g., excitons. In low-dimensional materials, Auger-Meitner (A-M) processes, density-dependent heat-generating exciton annihilation events, are prevalent even at modest exciton densities due to enhanced Coulomb interactions,^1^ and nonradiative pathways often dominate due to defects and natural background doping.^2,3^ The combination of these effects can lead to a significant fraction of absorbed light energy undergoing transduction to lattice heating, which coexists with other charge excitations like excitons. Similarly, in nanoscale electronic devices, charge carrier scattering with phonons leads to Joule heating and elevated device temperatures that impair efficient electronic dynamics, including transport, due to increased scattering with the lattice.^4^ As device dimensions and volume for heat dissipation continue to decrease, material interfaces increase, and carrier-boundary scattering also plays a key role in self-heating, limiting thermal and electrical conductivity.^5^ Each of these dissipative effects in optoelectronic devices is important to discern so that metrics such as the photoluminescent quantum yield (PLQY) and carrier diffusion length can be optimized. Additionally, thermal management strategies often leverage inherent material anisotropies in energy flow, which give rise to thermoelectric capabilities, the ability to reversibly convert an electric potential, *V*, to a temperature gradient, ∇*T*, or vice versa. In either case, distinguishing heat from charge when they coexist in a material and discerning their distinct photoinduced dynamics is vital not only for informing design principles for directing heat and charge in emerging materials but also for drawing well-informed conclusions about intrinsic material properties.

Distinguishing between heat and charge with optical measurements is, however, challenging. For example, both ground state and transient excited state optical spectroscopy manifest complex perturbations to the location, amplitude, and width of electronic resonances.^6−8^ Even if signatures of specific excitations are quantitatively observed, because their signatures can overlap spectrally and temporally, they remain difficult to quantitate despite judicious choices in excitation and probing wavelengths, time-dependent signatures, measuring as a function of voltage bias, and measuring a pump fluence or temperature dependence. In particular, local heating could influence or masquerade as electronic excitations in semiconducting materials, and because it is spectrally ubiquitous, isolating the electronic dynamics is especially challenging. Physically, heating leads to increased lattice fluctuations, which change the mass density, an effect measured by the thermo-optic coefficient and which may be observed as a frequency-independent change in the dielectric function.^9,10^ Near an electronic resonance, however, heating broadens and shifts the resonance feature, an effect that adds to or cancels any transient photoinduced changes from the electronic carriers themselves.^7,8^ This behavior is especially complicated in transition metal dichalcogenides (TMDCs) where heat dissipation following photoexcitation is prevalent and different exciton resonance features may spectrally overlap.^11^

The growing numbers of spatiotemporally resolved optical microscopies are excellent candidates for characterizing photogenerated energy and its transport in semiconducting materials because they provide spatial dynamics in addition to the information provided by more traditional time-resolved spectroscopy. These techniques, e.g., microtime-resolved photoluminescence (microTRPL),^12−15^ transient absorption microscopy (TAM),^16−18^ variations of transient reflectance microscopy,^19−21^ including stroboscopic scattering microscopy (stroboSCAT),^22^ directly measure the progressive expansion of initially localized populations of impulsively photogenerated energy carriers with nanoscale sensitivity and down to ultrafast time resolution. TRPL is, however, restricted to detecting electron–hole radiative recombination, and to the best of our knowledge, there are no reports of TAM separately resolving heat directly in addition to charge. Fortunately, due to its high sensitivity to changes in the real part of the dielectric function, even beyond transient reflectance alone, stroboSCAT has investigated heat flow in metallic composite films^23,24^ and has distinguished heat and charge flow in p-doped silicon based on different signs of imaging contrast, a substantial separation in time scales, and corroboration with commonly cited respective diffusivities.^22^ To address the additional challenges associated with characterizing electronic dynamics and transport in ultrathin semiconductors, especially when heat and electronic diffusivities are not as dissimilar as in silicon and where photogenerated heat is prominent, additional strategies must be developed.

Here, we directly co-measure both heat and excitons in few-layer molybdenum disulfide (MoS_2_) on relevant nanometer and picosecond length and time scales using stroboSCAT. Through a combination of near- and far-from resonant stroboSCAT probing conditions, one of which isolates heat alone, and calibration with steady state temperature-dependent reflectance contrast spectroscopy, we observe and quantitatively discern transient thermal and electronic contributions to the photogenerated dynamics. This strategy that we introduce enables isolation and characterization of the excitonic dynamics without concern for mistaking thermal contributions for electronic ones. This capability with few-layer MoS_2_ complements the capabilities of microTRPL, which is largely restricted to monolayer TMDC measurements^13−15^ because additional layers lead to very low PLQY due to the emergence of an indirect band gap. Furthermore, we corroborate our results with a spatiotemporal model for heat and exciton populations. With the ability to detect temperature elevations as low as 100 mK, our study suggests that even a modest temperature elevation has a substantial effect on the optical response in few-layer MoS_2_. More broadly, this work establishes a strategy for isolating electronic dynamics and transport in a wide range of conventional and emerging semiconductors that offers the exciting possibility of more incisively investigating thermal management and thermoelectric energy conversion.

To demonstrate the capability of identifying and simultaneously tracking electronic and thermal energy evolution, we focus on a hBN-encapsulated four-layer (4L) MoS_2_ flake fabricated using the hot pick-up technique and supported by a glass substrate.^27^ Before proceeding with spatiotemporally resolved transport measurements, we precharacterize the sample. The relevant portion of the electronic structure is shown schematically in Figure 1a.^25^ Optical reflectance microscopy readily identifies the encapsulated 4L region outlined in yellow in Figure 1b. Additional sample thickness characterizations are found in Figure S1. The steady state photoluminescence spectrum in Figure 1c shows characteristic strong emission from the spin–orbit split A (∼1.8 eV) and B (∼2 eV) direct excitons, with a strong A:B photoluminescence intensity ratio and dispersive resonance amplitude ratio in the blue reflectance contrast spectrum indicative of good sample quality.^28^ Photoluminescence due to recombination of the lowest-energy indirect exciton (IDE) in the near-infrared (∼1.4 eV), indicated by the blue arrow in Figure 1a, is comparatively weak.

**Figure 1:**
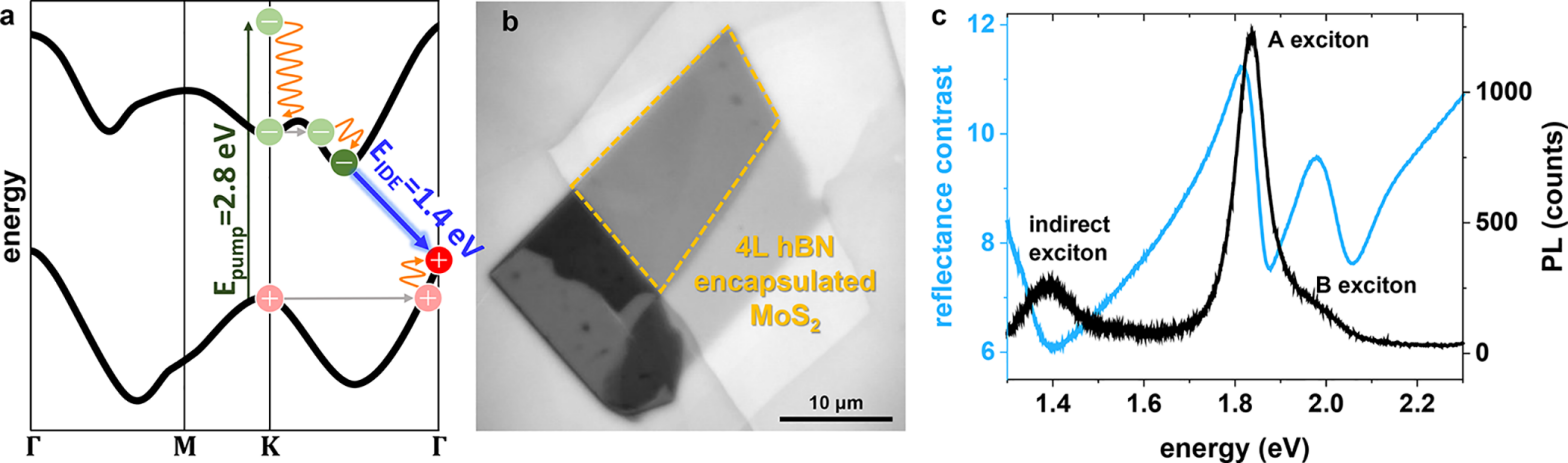
(a) Calculated single particle band structure for 4L MoS_2_ from ref (25) showing the above-band gap pump excitation (green arrow) at the K-point of the Brillouin zone,^26^ followed by fast thermalization (orange) and intervalley scattering (gray) to the lowest energy indirect exciton (blue arrow). (b) Optical reflectance image of the entire MoS_2_ flake with the measured 4L hBN-encapsulated region outlined in yellow. (c) Steady state photoluminescence (PL) spectrum (black) showing strong emission from the direct A exciton near 1.77 eV with a blue shoulder corresponding to emission from the direct B exciton. Emission from the indirect exciton (1.4 eV) is comparatively weaker. The reflectance contrast spectrum (blue) exhibits dispersive resonance features near the PL peaks.

We use stroboSCAT to directly visualize heat and exciton transport in 4L MoS_2_. stroboSCAT is a differential pump–probe technique that leverages the sensitivity of optical scattering to image energy migration. This sensitivity arises from homodyne detecting even the high angle scattered field via interference with a reference, in this case specularly reflected probe light. The physical process is similar to transient reflectance spectroscopy, except for the important difference that it takes place in a microscope with a wide-field—and often highly off-resonant—probe pulse capable of amplifying the signal that scatters off a more localized pump-induced excitation distribution.^22^ These particular experiments have nanometer spatial precision and ∼200 ps time resolution. Two pulsed diode laser sources are directed into the sample with a high numerical aperture microscope objective that also collects the signal and directs it to a CMOS camera (Materials and Methods). First, a near-diffraction-limited focused pump pulse of 2.8 eV photons (green arrow in Figure 1a) generates a Gaussian spatial distribution of photoexcitations in the sample, which are then probed after a controllable time delay by a single-wavelength widefield laser pulse of either 2.4 or 1.8 eV. Specifically in 4L MoS_2_, the pump pulse generates excitations at the K-point of the Brillouin zone.^26^ Efficient sub-ps intervalley scattering (horizontal gray lines in Figure 1a) and ∼fs phonon-assisted relaxation to the band edge (orange in Figure 1a) occur within the ∼200 ps experimental instrument response function (IRF), and we therefore expect the IDE to be the dominant electronic excitation on the time scale of our measurements.^16^ A differential signal image is generated through the difference of the image at a time delay after the pump and an image taken without a pump, normalized to the latter. The contrast in the image, (*R*_pump on_ – *R*_pump off_)/*R*_pump off_, is commonly referred to as Δ*R*/*R*. Despite the fact that 4L MoS_2_ is not luminescent, the presence of photoexcitations modifies the material’s local dielectric function, generating transient contrast that evolves as a function of space and time according to the quantity and location of decaying and diffusing photoexcitations. Energy population dynamics are described by integrated population decays as a function of time. More importantly, transport is characterized via the mean squared expansion (MSE) of the population in space as a function of time: MSE = σ^2^(*t*) – σ^2^(0) = 2*Dt*, where *D* is the diffusivity and σ(*t*) is the width of the Gaussian population at time delay, *t*, assuming a cylindrically symmetric distribution. In principle, any photoexcitation, e.g., charge carriers, excitons, phonons, may be detected in this way since the measurement observable, elastically backscattered light, does not rely on the material absorbing or emitting light at a particular frequency. Furthermore, near an absorption resonance, the sign of the differential contrast can be tuned above (bright) or below (dark) the baseline (gray) background. For a dispersive optical resonance, the transient response leads to oppositely signed Δ*R*/*R* on either side of the resonance.^29^ Heat, which also modulates electronic resonances, may also modify Δ*R*/*R*.

To isolate the effect of heating on the optical response, steady state reflectance contrast (RC) spectra^30^ are measured at a range of temperatures from room temperature to 90 °C, as described below and in the Materials and Methods. RC spectra are obtained by measuring reflected spectra from the sample atop of the substrate (*R*) and separately under the bare glass substrate (*R*_0_) and calculating *RC* = (*R* – *R*_0_)/*R*_0_. RC spectra over the measured temperature range are presented in Figure 2a, showing the characteristic A and B exciton resonance peaks red-shifting and broadening with increasing temperature. From these spectra, we identify two spectral regimes, near- and far-from resonance, which tune the relative contribution to the stroboSCAT signal from heat and excitons, enabling distinction between the two, as described below. (Although features from the shifting electronic resonances of the A and B exciton dominate the spectra in the visible regime, the lowest-energy IDE carries most of the excited state population on our measurement time scales.) We select probe energies for spatiotemporal imaging from available discrete laser diode sources indicated by the red (near resonant, 700 nm or 1.77 eV) and green (far-from resonant, 515 nm or 2.41 eV) vertical lines in Figure 2a.

**Figure 2:**
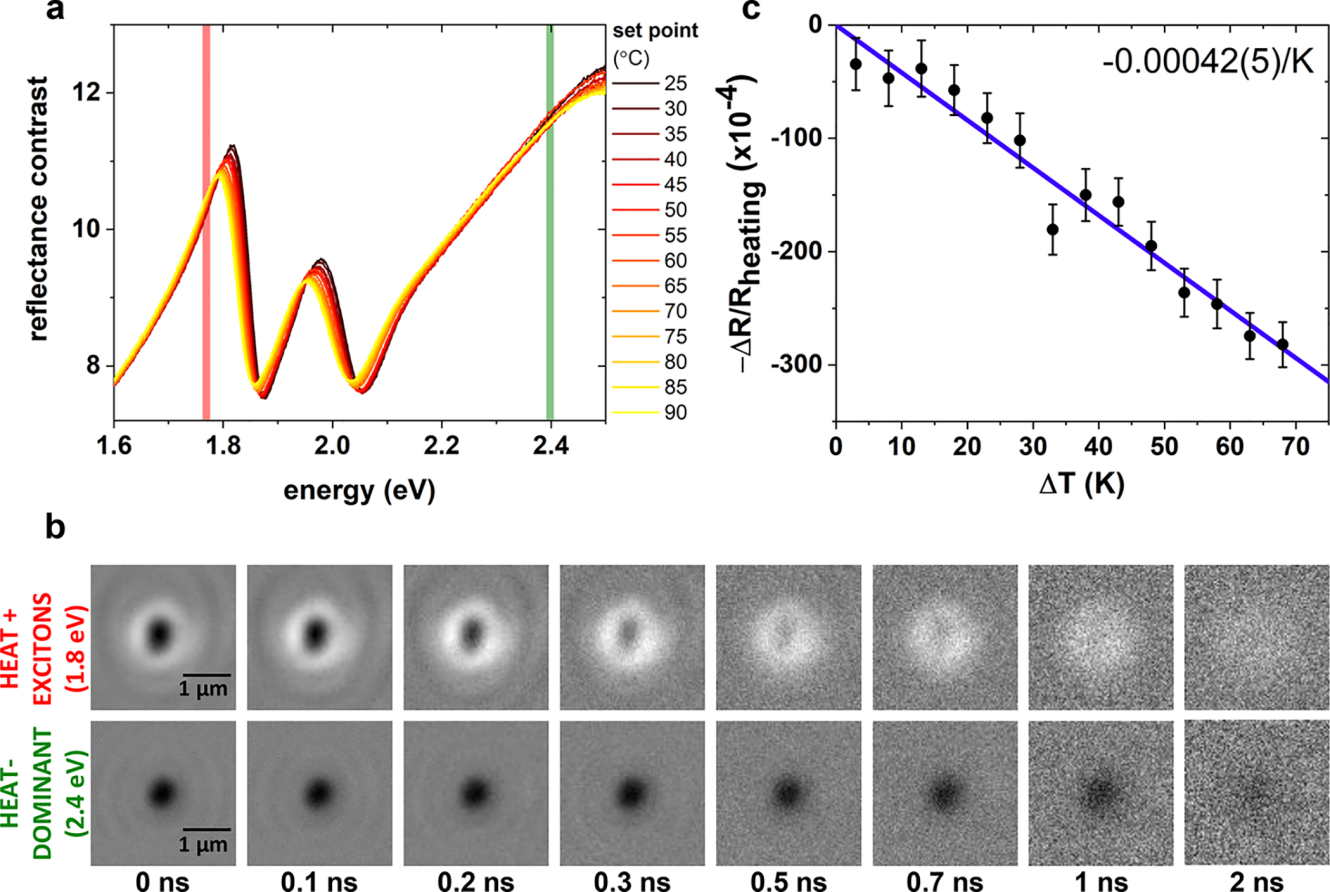
(a) Temperature-dependent reflectance contrast spectra over a range of temperature set points near the A and B exciton resonances. The near- and far-from-resonant probe energies are indicated with red and green lines, respectively. (b) stroboSCAT time series captured with a near-resonant (top) and far-from-resonant (bottom) probe. The focused 2.8 eV pump generates a peak initial exciton density of 3.5 × 10^13^ cm^–2^. (c) Expected differential contrast due to heating, , at 1.77 eV. The vertical axis is multiplied by −1 for direct comparison to widefield stroboSCAT measurements. The linear fit (blue line) has a fixed intercept through the origin. Error bars include contributions from the propagated standard error of the mean from averaged spectra, calculated reflectance contrast over the 4 nm laser line, and fit error to the vertical intercept in Figure S7.

## Results and Analysis

In the same sample region, we collect two complementary stroboSCAT measurements over a 7 ns time window by probing at the near- and far-from resonant energies (Figure 2b). In both measurements, the same applied pump pulse fluence of 35 μ J/cm^2^ generates an estimated peak exciton density of 3.5 × 10^13^ cm^–2^ (see Supporting Information), falling in an intermediate density regime where A-M interactions play a significant role but still being an order of magnitude below the Mott transition at which excitons would dissociate.^31^ Hot excitons thermalize and scatter to the indirect band edge within the IRF, transferring their excess energy to the lattice via efficient phonon emission. We therefore expect to observe both long-lived (several nanoseconds) IDEs and lattice heating simultaneously on our measurement time scales. In the far-from resonant stroboSCAT measurement, we observe negative (dark) contrast alone that decays and expands over several nanoseconds (Figure 2b, bottom), whereas in the near-resonant stroboSCAT measurement at the top of Figure 2b, we observe bright positive contrast beyond a dark, negative-contrast center that similarly decays over several nanoseconds, with positive contrast dominating after 1 ns. This contrast trend (negative contrast only when probing at 2.4 eV, positive and negative contrast when probing at 1.8 eV) and associated population dynamics and diffusivities persisted in multiple measured spots within the same sample and also in additional few-layer samples (Figure S3). We assume that each sign of contrast, positive or negative, in each measurement is generated by either heat or excitons, the two dominant forms of energy in the material following photoexcitation. Temperature-dependent RC spectra predict negative differential contrast due strictly to heating in the near-resonant measurement (Figure 2c and details below); therefore, we deduce that positive contrast in the same measurement must be due to the presence of excitons. Using these assignments, we observe that excitons diffuse faster than heat, giving rise to positive amplitude extending beyond the heat-dominant negative contrast. This assignment is consistent with previous reports of exciton and heat diffusivity in MoS_2_, in which exciton diffusivities are up to a few cm^2^/s^32,33^ while reported heat diffusivities are slower at ∼0.2 cm^2^/s.^34^ We note that these experiments are sensitive only to in-plane transport as the sample would need to be at least 30 nm, or ∼50 layers, thick in order to accumulate an interferometric phase flip that could indicate out-of-plane transport.^22^

**Figure 3:**
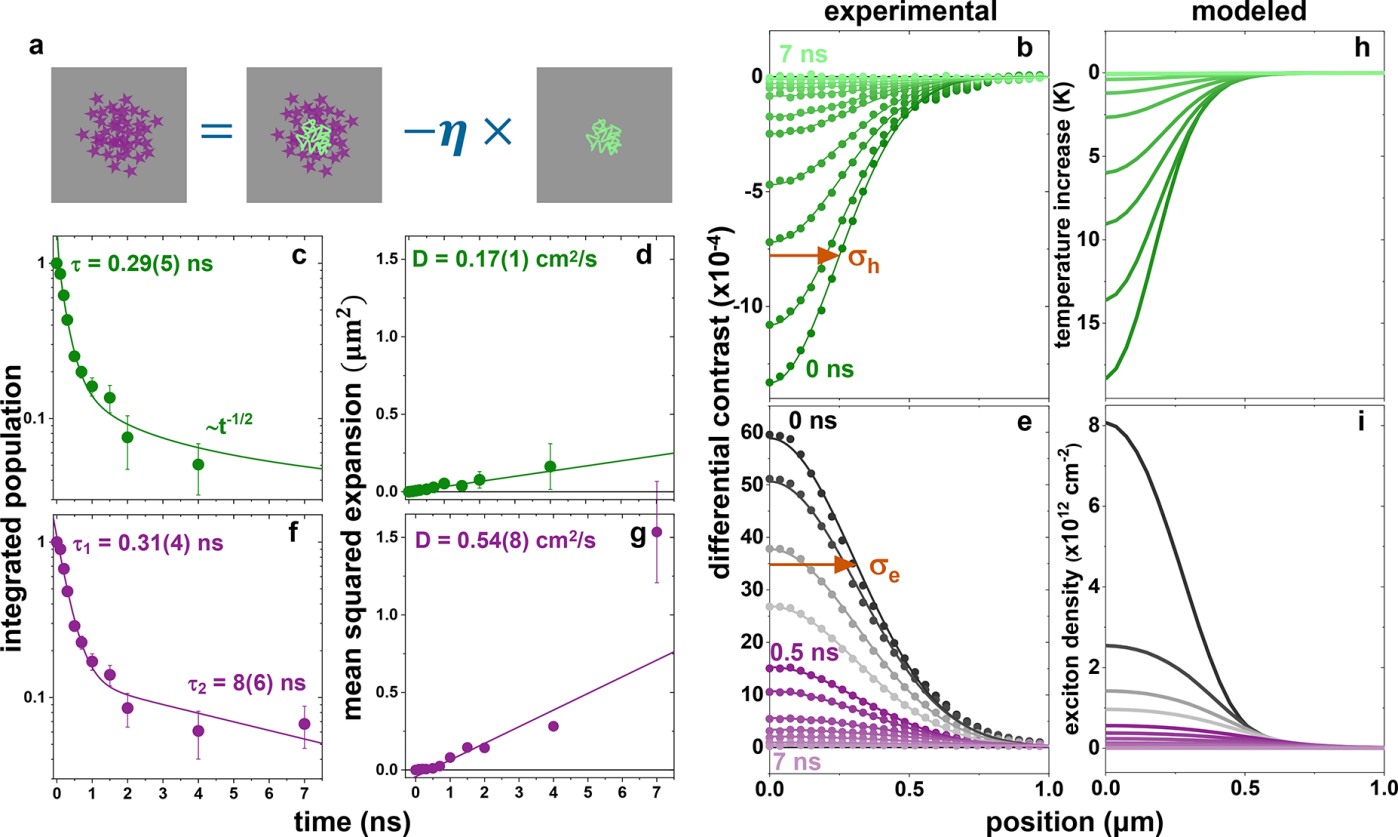
(a) Image arithmetic to isolate exciton distributions (purple) from thermal ones (green) when they coexist, with a scaling constant accounting for the wavelength-dependent sensitivity to optical perturbations depending on proximity to electronic resonances. (b) Azimuthal averages for the data shown in the bottom row of Figure 2b representing thermal distributions. (c, d) Integrated population decay fit to a single exponential plus power law (c) and mean squared expansion fit to a line (d) for the thermal distributions in (b). (e) Azimuthally averaged isolated exciton profiles after scaled image subtraction of the near- and far-from-resonant data sets in Figure 2b. (f, g) Integrated population decay fit to a biexponential (f) and mean squared expansion fit to a line starting at 0.5 ns (g) for the exciton distributions in (e). (h, i) Predicted thermal (h) and exciton (i) distributions from a spatiotemporal kinetic model best fit of the experimental data in (b, e). Orange arrows indicate the Gaussian fit widths to the time-zero population profiles for excitons and heat with σ_e_ = 0.3 μm and σ_h_ = 0.2 μm.

To most readily compare the data sets at the two imaging wavelengths, we apply a pixel size correction to account for their different point spread functions (PSFs) (Figure S4). With this correction, the spatial extent of the positive signal measured with the near-resonant probe is demonstrably larger than the corrected negative signal measured with the far-from resonant probe (Figure S5). This observation suggests that distinct dynamics give rise to the differing spatial extent of the positive and negative contrast signals probed near and far from resonance, respectively, furthermore confirming that they represent distinct photoexcited species. As the positive contrast has already been assigned to an exciton population, we deduce that the negative contrast far-from resonance is dominated by heat. Based on the probe spectral proximity to electronic resonances in the system, we estimate that the excitonic contribution far-from resonance is suppressed by a factor of 25 relative to the near-resonant exciton contribution. Furthermore, a single Gaussian function fits all far-from resonant data well; therefore, we deduce that the measurement is dominated by the optical response due to heating, and any potential contribution from excitons (positive or negative) is below our detection limit. It is therefore possible to isolate the positive exciton contribution to the near-resonant data set despite its spatial overlap with the negative heat contribution by subtracting the far-from resonant heat-dominant measurement using “image arithmetic” (Figure 3a). This strategy requires quantitative knowledge of the difference in the strength of the optical response due to heating at the two probe energies, the magnitude of which depends on the proximity to exciton resonances. We introduce a scaling factor, η, into this image subtraction to account for this difference: η = [strength of optical response due to heat at 1.8 eV]/[strength of optical response due to heat at 2.4 eV]. The far-from resonant stroboSCAT measurement characterizes the optical response due to heating at 2.4 eV. To quantitate the optical response due only to heating near resonance at 1.8 eV, we refer to the temperature-dependent reflectance contrast spectra in Figure 2a. To reframe the RC in units of stroboSCAT contrast, we calculate the difference in reflectance contrast at 1.8 eV found at each given elevated temperature and the value measured at room temperature and normalize it to the room-temperature response, Δ*R*/*R*_heating_ (Figures S6 and S7). The result is plotted in Figure 2c for each heater set point up to 90 °C (Δ*T* = 70 K) with a linear fit, the slope of which, −0.00042(5)/K, quantifies the predicted differential stroboSCAT contrast associated with a given temperature increase. To estimate the temperature at which to compare to η, we use a spatiotemporal kinetic model, described in more detail below, to iteratively fit exciton and heat experimental profiles until the predicted maximum achieved temperature in the MoS_2_ sample matches the value of η used in the fit. With this method, we estimate that the sample reaches a maximum temperature of 18 K above room temperature after accounting for fast interfacial heat transfer to surrounding hBN, a reasonable estimate that is close to the predicted temperature increase from thermalization to the band edge (see Supporting Information). The scaling factor η is therefore given by the ratio between the predicted maximum stroboSCAT contrast due to heat near resonance divided by the maximum stroboSCAT contrast at time zero in the far-from resonant measurement: .

With the interpretation and strategy developed above, we obtain the population dynamics and transport parameters for both heat and excitons in our encapsulated 4L MoS_2_. We determine the thermal dynamics by fitting each azimuthally averaged spatial distribution of each time point in the far-detuned stroboSCAT measurement to a Gaussian function (Figure 3b). We find that the integrated temperature profile has an initial fast ∼300 ps decay due to interfacial transfer to the encapsulating hBN, and then heat transfers more slowly, limited by the rate of heat diffusion in the hBN (Figure 3c). Although hBN is a good thermal conductor, its capacity to sink heat generated in MoS_2_ is limited by the evolving temperature gradient between the two materials and the finite volume of hBN, and since the amplitude of the Gaussian temperature distribution in the hBN drops due to lateral heat diffusion as *t*^–1/2^, this scaling determines the rate-limiting thermal transfer for the MoS_2_ in the ∼ns time frame (see Supporting Information). We obtain an in-plane heat diffusivity in the MoS_2_ of 0.17 cm^2^/s, consistent with the reported lateral thermal conductivity for MoS_2_ (Figure 3d).^34^

In order to quantitatively discern exciton dynamics from heat dynamics, we developed a careful strategy of frame-by-frame azimuthal profile subtraction of the near- and far-from resonant stroboSCAT data sets (Figure 3a). First, each Gaussian fit profile representing the heat population (temperature profile) in the far-detuned measurement is width- and amplitude-adjusted by a time-dependent PSF correction factor. This operation generates the *shape* of the isolated heat distribution that would have been measured with the near-resonant probe. Next, we multiply the PSF-corrected heat profiles by the above-deduced scaling factor, η, to quantitatively represent the differential contrast profile due to heating near the resonance (Figure S5). Finally, we azimuthally average the total near-resonant stroboSCAT signal and subtract the PSF-corrected and scaled thermal component obtained from the far-from resonant data set. The isolated radial exciton profiles are near-Gaussian, as shown in Figure 3e. The spatially integrated exciton population dynamics fit best to a biexponential exciton decay, which we attribute to density-dependent A-M interactions that dominate at early time delays when exciton densities are higher (τ_1_ = 310 ps), followed by slow nonradiative recombination of the IDE over τ_2_ ≈ 8 ns (Figure 3f). The exciton profiles at time delays earlier than 500 ps (grayscale) do not appear to expand, presumably due to the pump-induced profile and its early time changes being beneath the spatiotemporal resolution of these measurements. The extracted exciton diffusivity for ≥500 ps, once it is possible to see the profiles expanding, is 0.5 cm^2^/s, in agreement with other measurements in few-layer TMDCs (Figure 3g).^35,36^ We note that excluding the final 7 ns data point from the linear fit does not change this result within the fitting error. Repeating this dynamical analysis over a range of values of η from 1.4 to 7 enables an estimate of the uncertainties in the extracted diffusivities based on uncertainty in the initial maximum sample temperature elevation that we can safely bound between 4 and 20 K.

We support these findings with a spatiotemporal kinetic model that describes the coupled dynamics of excitons and heat. Additional details are included in the Supporting Information. We developed a simple set of coupled equations to capture the expansion and decay of excitons whose energy is overwhelmingly (due to very low PLQY) converted to heat via either nonradiative hot carrier relaxation following optical excitation or A-M decay:^13,37^
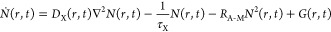
1
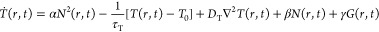
2Equation 1 describes the evolution of the exciton population, *N*(*r*, *t*). The first term describes exciton diffusion due to the pump-induced exciton population gradient with an exciton diffusivity of *D*_X_. The second term describes single exciton recombination, where τ_X_ is the recombination lifetime. The third term describes biexciton recombination due to A-M interactions, where *R*_A-M_ is the A-M coefficient. Finally, *G*(*r*, *t*) describes the generation of excitons from the pump pulse, which has a temporal pulse width of 72 ps and a spatial width, σ, of 168 nm. Equation 2 describes the temperature or heat population, *T*(*r*, *t*). The first term accounts for the temperature increase due to nonradiative relaxation of hot excitons that are created via A-M recombination, where , *E*_G_ is the indirect band gap energy, and *c* is the specific heat. The second term describes the decay of the temperature profile back to its initial room temperature value, *T*_0_, with a lifetime τ_T_, and the third term describes heat diffusion with diffusivity *D*_T_. The fourth term describes the temperature increase due to nonradiative single-exciton recombination, where . The last term describes the heat generated when excitons relax to the band edge after pump excitation, where  and *E*_P_ is the pump pulse energy. In addition, when propagating these equations in time, the heat decay phenomenologically transitions to a *t*^–1/2^ scaling after 700 ps to represent thermal transfer to hBN discussed in the Supporting Information. To fit the model to the time series of exciton profiles extracted above, along with the corresponding heat profiles obtained far-from resonance, we allow τ_X_ and *D*_X_ to vary only within the experimentally determined uncertainty and *R*_A-M_ to vary within the literature estimates.^38^ We approximate the indirect band gap by the peak position of the IDE PL (1.4 eV). All other parameters are fixed by our experimentally obtained values or from literature values.^26,39^

Figure 3h,i and Figure S8 show excellent qualitative agreement between the modeled and experimentally obtained exciton and thermal profiles. The model predicts an initial maximum exciton density of ∼8 × 10^12^ cm^–2^, lower than our experimental estimate based on the pump fluence and sample absorbance. We expect that this discrepancy arises because the model accounts for the finite pump duration and subsequent exciton decay occurring within the experimental IRF. (If we suppress A-M interactions, exciton decay, and exciton expansion in the model, we recover the initial condition for *N*(0, 0) = 3.5 × 10^13^ cm^–2^.) The predicted maximum temperature at experimental time-zero (predominantly from exciton thermalization after above band gap excitation) is 18 K, which is the value that is consistent with η of 6.1 (see Supporting Information). The best fit *R*_A-M_ coefficient, a parameter we cannot constrain with our experiments due to the experimental IRF, is 2.6 × 10^–3^ cm^–2^, which is lower than for monolayer TMDCs, but still within the expected range for multilayer TMDCs.^14,38,40^ Overall, the model supports our experimental finding that excitons diffuse slightly faster than heat, importantly enabling spatial differentiation between the two, which we described as being instrumental in the differential contrast assignment.

## Discussion

Having described our observations and analysis strategies and corroborated our results with simulations, we turn to a discussion of our findings and what they reveal and implicate. Below, we first establish the consistency of our physical model with other transient microscopy results that indirectly reveal an impact of heat on exciton populations in 2D TMDCs. Second, we explore stroboSCAT’s favorable temperature sensitivity, which, under the conditions employed in this work, is as good as ∼100 mK. Third, we explore the value in employing our spatiospectrotemporal approach and discuss complementary, related strategies that could be used as a general toolkit to draw from, depending on the particular photophysical details of any given material, given the ubiquity of heat generation. Finally, we suggest strategies to treat increasingly complex combinations of energy carriers and point toward the utility of our approach in elucidating and leveraging mechanisms of electron–phonon coupling in thermal management and thermoelectrics.

To test our model-related findings, we note that Perea-Causín et al.^37^ also employed transient optical microscopy to study the interplay between heat and excitons in 2D TMDCs, albeit with different experimental parameters. When registering the exciton photoluminescence of monolayer WS_2_, following ultrafast pump excitation leading to a far greater nonequilibrium-exciton effective temperature elevation, they observed “halo-like” spatial photoluminescence profiles, which they attributed to radially outward excitonic transport driven by strong temperature gradients. To relate our model to these results, we added the Seebeck term , as in Perea-Causín et al.,^37^ to eq 1 to reflect that exciton diffusivity is driven not only by exciton density gradients but also by thermal ones. Here, σ(*r*, *t*) = *N*(*r*, *t*)*qμ* is the electric conductivity for exciton density *N*, elementary charge *q*, and exciton mobility, μ. *S* is the Seebeck coefficient and is an intrinsic material parameter. By using the Perea-Causín parameters in the model, we indeed generated halo-shaped excitonic profiles (Figure S9). If, however, we retain the Seebeck term to attempt to fit our experimental results that have a less impulsive pump pulse and furthermore allow η to vary, we find that an exceedingly large Seebeck coefficient (*S* > 10,000 V/K) would be required to generate halo-like exciton profiles. For this reason, we rule out this Seebeck regime to model our observations in 4L MoS_2_. This finding is also consistent with results from Zipfel et al.^14^, who also do not observe exciton halos in encapsulated samples with *R*_A-M_ ≲ 10^–3^ cm^–2^.

**Figure 4:**
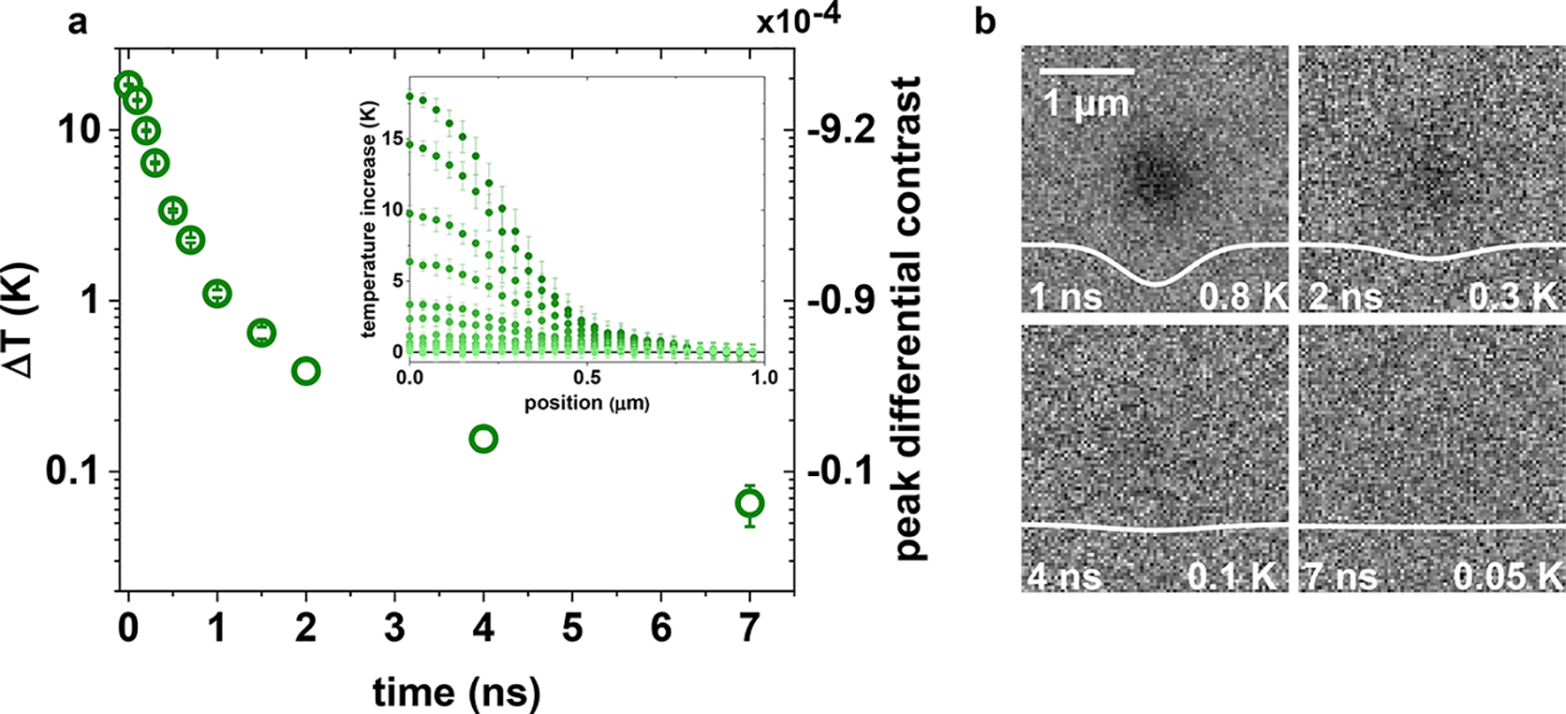
(a) Gaussian amplitude fit decay for the far-from resonant probe data set. The vertical axis is calibrated using the maximum predicted sample temperature elevation from the spatiotemporal model (18 K) and the measured maximum differential contrast value. The inset shows the azimuthal averages in Figure 3b with a rescaled vertical temperature axis. Error bars represent the standard error of the mean of the azimuthal averaging. (b) Far-from resonant probe images labeled with the maximum temperature achieved in each frame. All images are shown on the same contrast scale. White traces are Gaussian fits to the raw azimuthally averaged data.

We also wish to highlight a valuable byproduct of developing thermal sensitivity capabilities in a transient optical microscopy, namely that stroboSCAT can serve as a highly sensitive noncontact thermometry with excellent spatial resolution compared to infrared analogs. For example, based on the 18 K temperature elevation calculation in the 4L MoS_2_, we reframe in Figure 4 the relaxation of the temperature in Figure 2b and Figure 3c to establish the sensitivity of this thermometry. Figure 4a relabels the axis of Figure 3c with the time-dependent peak temperature at the center of the heat distribution. The radial profiles of corresponding time delays, also in Figure 3b, are shown as an inset, and some example Δ*R*/*R* images are included in Figure 4b. At the longer time delays, we thus establish the ability to resolve an ∼0.1 K temperature elevation. Although it is difficult to directly compare with the metrics of more conventional thermal imaging,^41^ this value seems on par with it and enjoys substantially higher spatial resolution. While the present data were collected by only averaging for 7 min per time point, we estimate, based on the 12-bit CMOS camera well depth and the shot noise limit, that our sensitivity should indeed be in the range of 100 mK. With increased averaging, microscope stabilization, and detector sensitivity, this limit could be pushed into the tens of mK regime, making stroboSCAT an exquisite thermometry approach with added high spatial resolution. Not only is this spatially resolved sensitivity to temperature that we have discovered very powerful for discerning heat and charge in photoexcited materials, but it should also find great utility in thermal management characterization in the semiconductor device sector.

Regarding the toolkit that we have developed and its more general applicability, our spatiospectrotemporal approach is able to characterize the complex, overlapping electronic and thermal system dynamics even though their contributions would be difficult to disentangle from spectroscopic data alone; discerning these dynamics in 4L MoS_2_ was possible despite an absence of strongly distinct time scales in the excitonic and thermal population dynamics. Another strategy to achieve the same result could be to use the temperature-dependence of the reflectance contrast to calibrate the response to heat at 2.4 eV, not only at 1.8 eV, where the proximity to a zero-crossing precluded this process in the current work. Careful selection of another far-from resonance probe wavelength could enable this alternative strategy for future work. As a general strategy, for each specific sample, one must carefully consider the best approach to isolate the electronic contribution to the photoexcited signal based on spectral information, as discussed here, and the extent to which the thermal and electronic diffusivities create a separation of time scales, which is minimal here but was, for example, sufficient to investigate silicon.^22^ Furthermore, there are generally multiple different processes by which heat is generated that can occur on different time scales relative to the generation and evolution of electronic excitation. Regardless, the impact of heat on transient measurements is substantial in many materials, not only for few-layer TMDCs.^7,42,43^ Any type of nonradiative process, from above band gap excitation, to nonlinear processes like annihilation, including Auger-Meitner effects, to “standard” nonradiative decay, will generate heat. By measuring the photoinduced response in a reflectance geometry, the thermal response is revealed most clearly due to its superior sensitivity to the real part of the dielectric function, relative to transmission-based (or photoluminescence) measurements. Again, while it has been painstakingly investigated in semiconductor transient spectroscopy,^7,8^ which is largely performed in reflectance geometry due to the opacity of semiconductors in the visible and near-infrared parts of the spectrum, the higher sensitivity and the addition of the spatial variable that stroboSCAT affords provide additional helpful constraints to discern heat and charge. The spatial coordinate is helpful not only because of access to instantaneous spatial distributions but also to the time rate of change in the spatiotemporal evolution, which yields transport parameters such as diffusivity.^44^ We therefore anticipate this ability to characterize the coexistence, transport, and interplay of heat and charge in materials to be highly general and to enable a more detailed and reliable mechanistic understanding of a material’s physical properties and phenomena far more broadly than in TMDCs alone.

Regarding strategies for the future, the possibility exists to interrogate materials with more than two types of energy carriers; for example, free carriers, trions, and excitons coexist together with one another and also with heat. In 4L MoS_2_, we probed sufficiently far from a zero crossing in the transient reflectance spectrum in all cases to avoid sign-changes in the differential contrast associated with each type of energy. In materials with additional distinct electronic species, the combination of measuring at additional pump energies and fluences to tune the relative densities of distinct photoinduced energy carriers could enable them to be distinguished. Furthermore, a continuously tunable probe source could be leveraged to increase the signal-to-noise ratio of photoexcitations that may not appreciably modify the local dielectric function.^45,46^

It should also be possible to measure both electronic and thermal energy transport in more heterogeneous material configurations as are found in various devices, especially when they are composed of organic semiconductors. Due to stroboSCAT’s differential nature, structural irregularities are effectively normalized away, leaving behind a clear view of how transport is impacted by them. If there are localized and well-separated subdiffraction defects or interfaces, their impacts on transport can be resolved, as we saw previously in ref (22), generating steps in the mean squared expansion. This can be employed to reveal anomalous diffusion of heat^24^ as well as charge. When defects or interfaces are more closely spaced, the observed transport properties will reflect the net effect of the constituent materials and interfaces. Nevertheless, transport measurements on individual material components and on more controlled interfaces may be used as control experiments to aid in explaining the aggregate heterostructure behavior. Furthermore, the impact of out-of-plane heterogeneities may be resolved by investigating phase changes in the stroboSCAT contrast or by considering the population dynamics observed in the presence of interfaces in addition to the lateral transport, similar to the way that we treat thermal transfer between MoS_2_ and hBN in this work or to the way that interfacial transfer is examined in ref (24).

We also envision a range of additional utilities of stroboSCAT in elucidating mechanisms of electron–phonon coupling and deepening our understanding of intrinsic thermal–electronic energy conversion and transport. Spatiotemporally monitoring charged photoexcitations and phonons simultaneously provides alternative possibilities for discovering mechanisms of electron–phonon scattering. In particular, understanding nonradiative decay pathways facilitated by traps, interfaces, defects, A-M interactions and natural background doping will elucidate design principles for engineering higher PLQY materials and directed or enhanced diffusion lengths.^47−50^ Characterizing the potential interplay between heating and electronic energy flow could inform thermal management strategies by revealing the dominant factors and mechanisms that tune electron–phonon coupling. Furthermore, while thermal management aims to mitigate the impact of unwanted and deleterious heat dissipation, for example, in semiconductor electronics, stroboSCAT also has the capability to directly measure transport anisotropies and thermoelectric effects in which heat is harnessed to do useful electronic work. For example, directly measuring the intrinsic Seebeck coefficient, an important factor in the intrinsic figure of merit for thermoelectrics, across different device configurations or material thicknesses may address challenges in efficiently upcycling heat loss through conversion to electricity or otherwise controlling heat flow in operating devices that suffer from poor performance due to self-heating.^51^ In particular, the interferometric depth-dependent stroboSCAT contrast in sufficiently thick layered van der Waals materials might be able to distinguish between out-of-plane transport and more rapid in-plane transport facilitated by strong covalent bonding, which is a potentially important design parameter for thermoelectric devices.

## Conclusion

In conclusion, we use a combination of optical scattering microscopy and temperature-dependent reflectance contrast spectroscopy to co-measure and discern the photoinduced dynamics of heat and excitons in 4L MoS_2_. This capability is a generalizable consequence of stroboSCAT’s spatially, spectrally, and temporally resolved contrast mechanism that is sensitive to any perturbation that modifies a material’s local dielectric function. The spatiotemporal energy maps play a key role in identifying overlapping energetic populations with distinct contributions to the differential contrast. Our results agree with previous characterizations of few-layer MoS_2_, are robust to experimental uncertainties in the estimated sample temperature elevation, and demonstrate a temperature sensitivity as good as 100 mK, ushering in an era for spatiotemporally resolved optical microscopy to discern charge and heat and their potential interplay.^52^ With quantitative energy-carrier-specific tracking down to few-picosecond time scales, provided structural heterogeneities are sufficiently spaced for the distinct transport properties they produce to be spatially resolved, directly characterizing and explaining the factors that give rise to the optoelectronic properties of a wide range of emerging semiconducting materials, including intrinsic thermoelectrics and low-dimensional or organic electronic devices, is now possible without having to rely on complex models or assumptions.

## Materials and Methods

### MoS_2_ Preparation and Characterization

We use the hot pick-up technique to fabricate hBN-encapsulated few-layer MoS_2_ heterostructures on coverglass.^27^ Briefly, hBN and MoS_2_ are exfoliated onto 285 nm of thermally oxidized SiO_2_ on Si. We made stamps consisting of PDMS covered with a thin film of the thermoplastic polymer polycarbonate (PC). Using these stamps, we first pick up the top hBN, then the desired MoS_2_ flake, and then the bottom hBN, all at 50 °C. We deposit the stack onto #1.5 coverglass, which serves as the substrate for all stroboSCAT measurements, at 180 °C, and then dissolve the PC in chloroform.

We characterized the hBN-encapsulated MoS_2_ sample using optical microscopy, Raman spectroscopy, and atomic force microscopy (AFM). The 4L MoS_2_ flake measured in the main text is outlined in blue in Figure S1a,b. The AFM image in Figure S1b shows that our sample has large (>7 × 7 μm), homogeneous, bubble-free areas, which are ideal for measuring in stroboSCAT. The separation between the two MoS_2_ Raman peaks demonstrates that these samples are 4 layers thick Figure S1c,d.^53^ The hBN layer between the MoS_2_ flake and glass substrate is thin enough (∼5 nm) to allow easy optical access to the MoS_2_ layer within the 1.4 NA objective’s depth of field.

### stroboSCAT Measurements

stroboSCAT measurements were performed at room temperature using three Picoquant pulsed laser diode sources, one to excite the sample at 440 nm (LDH-D-C-440) and two probe sources at 515 nm (LDH-D-C-520) and 700 nm (LDH-D-C-705). The base laser repetition rate was set to 10 MHz with the pump modulated at 660 Hz. Pump–probe time delays were electronically controlled by the laser driver with <20 ps precision. The pump and probe beams were spatially filtered through 25 and 50 μm pinholes, respectively, before being combined with a dichroic mirror (DMLP505, Thorlabs) and directed into the objective of an inverted microscope stage with a 50/50 beamsplitter. The pump was focused onto the sample with this high numerical aperture oil-immersion objective (Leica HC PL APO 63×/1.40NA) to a fwhm of ∼300 nm. The probe was focused into the back focal plane of the same objective to enable widefield illumination in the sample plane. The backscattered light was collected through the same objective, filtered (ThorLabs FB520-10 or Chroma ET720/60m) to reject the pump light, and focused onto a CMOS detector (PixeLINK PL-D752) using a 500 mm focal length imaging lens, resulting in an overall image magnification of 157.5×. The instrument response function was measured to be ∼240 ps, primarily limited by the pulse duration of the diode sources (60–110 ps). Extensive details of the stroboSCAT technique are described in previous work.^22^

Differential Δ*R*/*R* stroboSCAT images at each probe time delay are constructed from the difference between a widefield image collected after a pump pulse excitation and a widefield image collected without pump pulse excitation, normalized to the latter: Δ*R*/R = (*R*_pump on_ – *R*_pump off_)/*R*_pump off_. Each of these sequentially taken individual images is obtained within the 1.3 ms camera exposure. To construct the images in Figure 2c, a total of 3500 pairs of pump-on + pump-off image exposures at each time delay were collected in each of 10 scans over the full set of time delays. The results of each time delay from these 10 scans were averaged together for a total averaging time of 7 min per time-delayed differential image. Images are contrast-adjusted so that the grayscale baseline is in the center of the scale with the maximum signal magnitude defining both positive and negative scale bounds. The −5 ns time delayed image is subtracted from each subsequent time delayed image as a baseline correction. We performed similar measurements in the same sample > *rbin*10 times at 5 different pump fluences (5–85 μJ/cm^2^), yielding similar contrast and dynamical trends. Measurements in a separate 4L MoS_2_ sample also yielded similar results. In constructing these differential images with only 1.3 ms between each pump-on or pump-off image acquisition, many structural irregularities are effectively normalized away, leaving behind the well-defined edge features and homogeneous material regions imaged in this sample system.

### Temperature-Dependent Reflectance Contrast Spectroscopy

Broadband emission from a stabilized tungsten-halogen lamp source (ThorLabs SLS201L) is spatially filtered through an optical fiber and then focused into a 0.9 NA air objective in a Leica DMi8 inverted microscope, illuminating the sample within an ∼1 μm spot size. The reflected light output is fiber-coupled to a calibrated Princeton Instruments spectrometer (HRS300) with ∼1.5 nm spectral resolution. Spectra were collected by averaging 500 frames (10 ms exposure per frame) together after heating and equilibrating the sample using a PID-controlled metal ceramic heater (ThorLabs HT19R).

### Spatiotemporal Model

A set of coupled equations representing exciton and heat dynamics and transport and their interchange was recast in natural units and solved using the pdepe function in MATLAB. Multistart optimization was performed with 500 starting points over a constrained 3-parameter space with least-squares minimization to the measured heat and isolated exciton profiles. More details are provided in the Supporting Information.

## Supporting Information Available

Thickness characterization of encapsulated four-layer MoS_2_; additional stroboSCAT measurements in few-layer MoS_2_; transfer matrix calculations; point spread function correction; temperature-dependent reflectance spectroscopy and construction of differential contrast due solely to heating; maximum sample temperature estimate from calorimetry; long-time temperature decay scaling; spatiotemporal model; spatiotemporal kinetic model with Perea-Causín et al. experimental parameters (PDF)
