# Supplementary material for: Detecting, Distinguishing, and Spatiotemporally Tracking Photogenerated Charge and Heat at the Nanoscale
Source: ACS Nano. 2023 Sep 18;17(19):19011–21. doi: 10.1021/acsnano.3c04607 (PMC10569093; doi:10.1021/acsnano.3c04607)
Supplement: Supplementary file 1 — nn3c04607_si_001.pdf [file nn3c04607_si_001.pdf]

Supporting Information: Detecting, distinguishing, and spatiotemporally tracking photogenerated charge and heat at the nanoscale

Hannah L. Weaver,[†] Cora M. Went,[‡] Joeson Wong,[¶,∇] Dipti Jasrasaria,[§,††] Eran Rabani,[§,‖,⊥] Harry A. Atwater,[¶] and Naomi S. Ginsberg[*,§,†,#,‖,@,△]

†Department of Physics, University of California, Berkeley, CA 94720, United States
‡Department of Physics, California Institute of Technology, Pasadena, CA 91125, United States
¶Department of Applied Physics and Materials Science, California Institute of Technology, Pasadena, CA 91125, United States
§Department of Chemistry, University of California, Berkeley, CA 94720, United States
‖Materials Sciences Division, Lawrence Berkeley National Laboratory, Berkeley, CA 94720, United States
⊥The Sackler Center for Computational Molecular and Materials Science, Tel Aviv University, Tel Aviv, Israel 69978
#Molecular Biophysics and Integrated Bioimaging Division, Lawrence Berkeley National Laboratory, Berkeley, CA 94720, United States
@Kavli Energy NanoScience Institute, Berkeley, CA 94720, United States
△STROBE, NSF Science & Technology Center, Berkeley, California 94720, United States
∇Current address: Department of Chemistry, University of Chicago, Chicago, IL 60637
††Current address: Department of Chemistry, Columbia University, New York, NY 10027, United States

E-mail: nsginsberg@berkeley.edu

1. Thickness characterization of encapsulated four-layer MoS_2

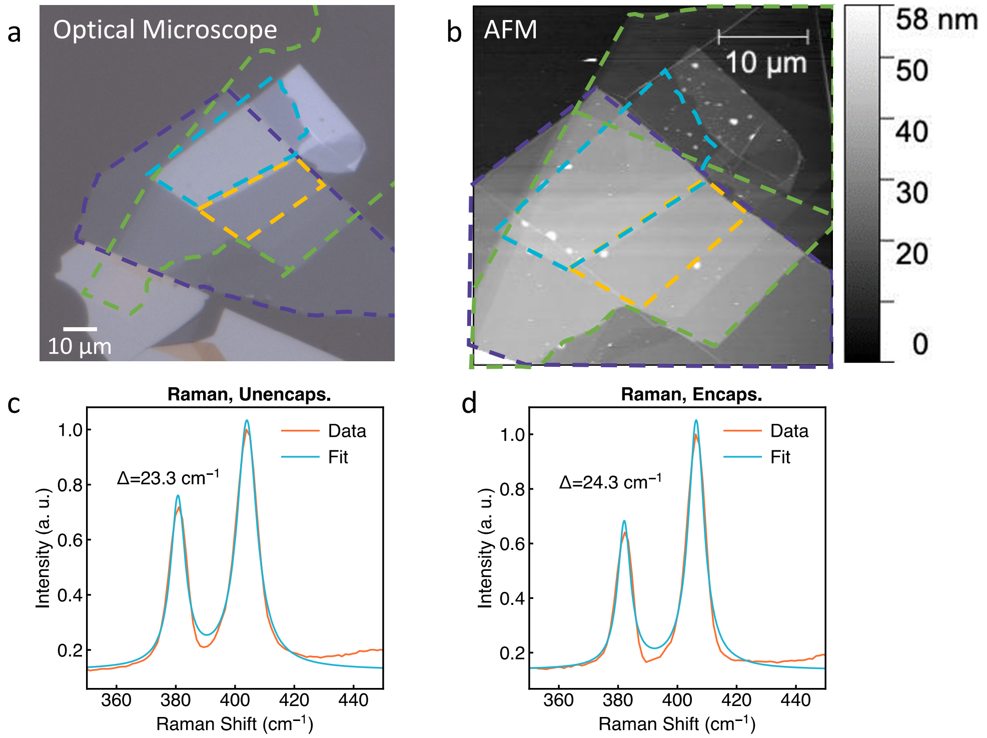

Figure S1: (a) Optical microscope image of hBN-encapsulated MoS_2 where blue outline is 4L MoS_2, yellow outline is 1L MoS_2, green outline is substrate-adjacent hBN and purple outline is air-adjacent hBN. (b) Atomic force microscopy scan of sample used to identify hBN thicknesses. (c, d) Raman spectroscopy of out-of-plane A_{1g} mode in pre-encapsulated (c) and post-encapsulated (d) region, demonstrating a shift that corresponds to an MoS_2 thickness of 4 layers.

2. Additional stroboSCAT measurements in few-layer MoS_2

We performed measurements in a separately prepared 4L MoS_2 sample with and without hBN encapsulation (Figure SS2a). While the positive contrast excitonic contributions are similar in amplitude for the same excitation density, the negative contrast heat contribution is far more substantial in the unencapsulated MoS_2, owing to the high thermal conductivity (heat sinking) of the hBN. This data comparison allows us to change only the heat contribution. As a result, we observe that the net signal's cross section in the figure changes from negative to positive values at a larger radius in the unencapsulated sample, as one would expect when adding a more substantial heat contribution to the same excitonic population (Figure SS2b).

We performed additional stroboSCAT measurements in 4L MoS_2 at varying pump fluences from 5-85 $\mu J/cm^2$ to confirm that the near- and far-from resonant contrast trends

persisted (Figure SS3).

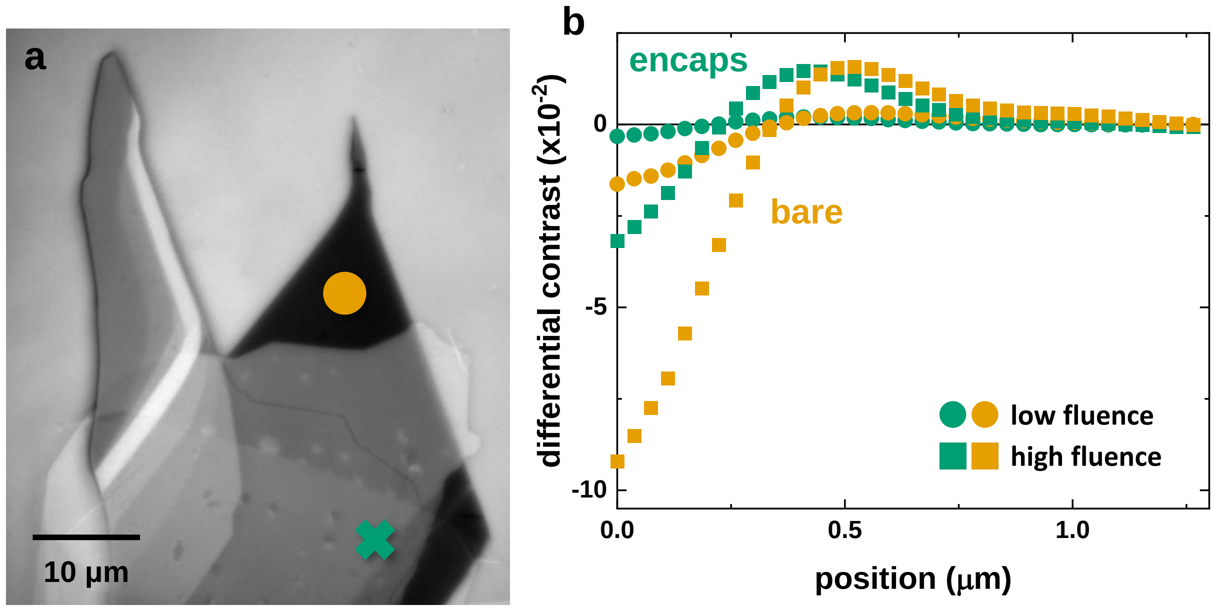

Figure S2: (a) Optical reflectance image of a separate few-layer MoS_2 sample with an hBN-encapsulated region (near green ex) and unencapsulated region (near gold circle). (b) Time zero radial profiles measured in each region, with "low fluence" corresponding to an exciton density of $\sim 2 \times 10^{13}$ cm^{-2} and "high fluence" corresponding to an exciton density of $\sim 10 \times 10^{13}$ cm^{-2}.

3. Transfer matrix calculations

We use transfer matrix calculations executed with the "tmm" Python software package[S1,S2] to estimate the total absorbance over the four layers of material in the sample, which is enhanced by multiple internal reflections and interference at the two hBN-MoS_2 interfaces. We input complex refractive index values from the literature for 4L MoS_2[S3] and a flat dispersion for hBN ($n_{\mathrm{hBN}} = 2.2$) with no absorbance in the visible ($k_{\mathrm{hBN}} = 0$).[S4] The input layer thickness of MoS_2 is 0.65 nm,[S5] while the hBN thicknesses are experimentally estimated with AFM (5 and 19 nm for the bottom and top layers, respectively). The calculation predicts that 6% of incident photons are absorbed per layer, resulting in an overall $1/e^2$ carrier density of 4.8×10^{12} cm^{-2}, or a peak carrier density of 3.5×10^{13} cm^{-2}. By contrast, a simple absorbance calculation using $\alpha(2.8\text{ eV}) = 7.2 \times 10^5$ cm^{-1} yields an estimated $1/e^2$ carrier density of 1.5×10^{12} cm^{-2}, or a peak carrier density of 9.3×10^{12} cm^{-2}, an underestimate by a factor of 3. The spatiotemporal model we describe in the main text and below estimates a maximum exciton density at time zero of $\sim 8\times10^{12}$ cm^{-2}, lower than that predicted by the transfer matrix calculation because it takes into account a finite pump pulse duration (72 ps) over which some exciton-exciton annihilation occurs. Furthermore, a binding energy of $\sim 4k_{\mathrm{B}}T$

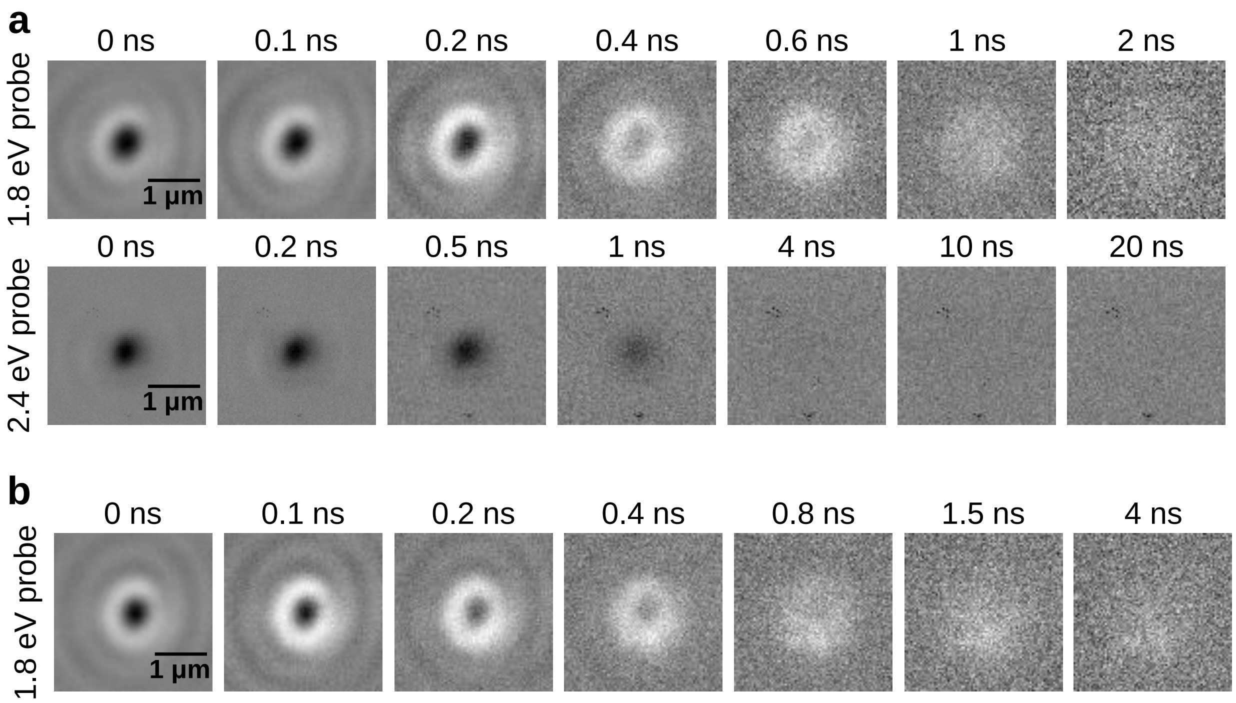

Figure S3: (a) Repeated stroboSCAT measurement at higher pump fluence (40 µJ/cm^2) in a different region of the sample measured in the main text. (b) stroboSCAT measurement at the encapsulated location marked in Figure S2a using the highest measured pump fluence (85 µJ/cm^2).

(at room temperature) suggests that dissociated free carriers make up $< 2\%$ of the total photoexcited electronic population.

4. Point spread function correction

A diffraction-limited point spread function (PSF) is well-approximated by a normalized Gaussian function:

$$\mathrm{PSF}_\lambda(r) = \frac{1}{2\pi\sigma_\lambda^2}\,\mathrm{e}^{-r^2/2\sigma_\lambda} \tag{1}$$

where $r^2 = x^2 + y^2$ and σ_λ is given by the Abbe diffraction limit:

$$\sigma_\lambda = \frac{\lambda}{2\ \mathrm{NA}\ 2\sqrt{2\ln 2}}. \tag{2}$$

In these experiments, excitons are generated by a nearly diffraction-limited 440 nm pump pulse and then efficiently convert to lattice heating, resulting in an initial Gaussian heat distribution:

$$G_{\mathrm{pop}}(r) = N_{\mathrm{pop}}\,\mathrm{e}^{-r^2/2\sigma_{\mathrm{pop}}^2} \tag{3}$$

where σ_{pop} is the width of the actual heat population (temperature profile). This distribution is imaged in one set of experiments by a widefield 515 nm probe with a PSF given by

$$\mathrm{PSF}_{515}(r) = \frac{1}{2\pi\sigma_{515}^2}\,\mathrm{e}^{-r^2/2\sigma_{515}^2} \tag{4}$$

where σ_{515} is defined by Equation 2. This imaging operation yields a measured Gaussian distribution, $G_{\mathrm{meas},515}(r)$, and may be represented by a 2D convolution:

$$G_{\mathrm{meas},515}(r) = G_{\mathrm{pop}}(r) \otimes \mathrm{PSF}_{515}(r) = \frac{N_{\mathrm{pop}}\sigma_{\mathrm{pop}}^2}{\sigma_{\mathrm{pop}}^2 + \sigma_{515}^2}\,\mathrm{e}^{-r^2/2(\sigma_{\mathrm{pop}}^2+\sigma_{515}^2)} = \frac{N_{\mathrm{pop}}\sigma_{\mathrm{pop}}^2}{\sigma_{\mathrm{meas},515}^2}\,\mathrm{e}^{-r^2/2\sigma_{\mathrm{meas},515}^2} \tag{5}$$

where

$$\sigma_{\mathrm{meas},515}^2 = \sigma_{\mathrm{pop}}^2 + \sigma_{515}^2. \tag{6}$$

From experimentally measured data at time-zero, $\sigma_{\mathrm{meas},515} = 180$ nm, so for a diffraction-limited microscope, the initial heat distribution, from Equations 2 and 6, has a width of $\sigma_{\mathrm{pop}} = \sqrt{\sigma_{\mathrm{meas},515}^2 - \sigma_{515}^2} \approx 160$ nm, which is slightly larger than the pump width of $\sigma_{\mathrm{pump}} \approx 130$ nm, to be expected since the heat distribution may expand slightly during the temporal overlap of the ~100 ps pump and probe pulses.

If it were possible to measure only heat with a 700 nm probe, the measured Gaussian distribution, $G_{\mathrm{meas},700}(r)$, would be represented by a different convolution:

$$G_{\mathrm{meas},700}(r) = G_{\mathrm{pop}}(r) \otimes \mathrm{PSF}_{700}(r) = \frac{N_{\mathrm{pop}}\sigma_{\mathrm{pop}}^2}{\sigma_{\mathrm{meas},700}^2}\,\mathrm{e}^{-r^2/2\sigma_{\mathrm{meas},700}^2} \tag{7}$$

where

$$\sigma_{\mathrm{meas},700}^2 = \sigma_{\mathrm{pop}}^2 + \sigma_{700}^2, \tag{8}$$

and again, σ_{pop} is the width of the actual generated heat population which, under identical excitation conditions, does not change with probe wavelength.

The goal is to transform the measured 515 nm heat distribution data to what would have been measured with a 700 nm probe in order to perform an accurate subtraction of the heat distribution from the total measured differential signal measured with 700 nm, which includes heat and excitonic contributions. The transformation of the measured 515 nm data for this purpose requires suppressing the amplitude and stretching the width of $G_{\mathrm{meas},515}(r)$ to represent the additional "smoothing" that would occur with a broader imaging PSF. To make this transformation more concrete, we define a correction factor, κ:

$$\kappa \equiv \frac{\sqrt{\sigma_{\mathrm{pop}}^2 + \sigma_{700}^2}}{\sqrt{\sigma_{\mathrm{pop}}^2 + \sigma_{515}^2}} = \frac{\sigma_{\mathrm{meas},700}}{\sigma_{\mathrm{meas},515}} \tag{9}$$

where $\kappa > 1$. We express Equation 7 in terms of κ and known variables. The amplitude of $G_{\mathrm{meas},700}(r)$ may be expressed as:

$$\frac{N_{\mathrm{pop}}\sigma_{\mathrm{pop}}^2}{\sigma_{\mathrm{meas},700}^2} = \frac{1}{\kappa^2}\frac{N_{\mathrm{pop}}\sigma_{\mathrm{pop}}^2}{\sigma_{\mathrm{meas},515}^2}, \tag{10}$$

and the Gaussian exponent of $G_{\mathrm{meas},700}(r)$ may be expressed as:

$$-\frac{r^2}{2\sigma_{\mathrm{meas},700}^2} = -\frac{1}{\kappa^2}\frac{r^2}{2\sigma_{\mathrm{meas},515}^2} = -\frac{(r/\kappa)^2}{2\sigma_{\mathrm{meas},515}^2} = -\frac{r'^2}{2\sigma_{\mathrm{meas},515}^2} \tag{11}$$

where $r = \kappa r'$ represents the "contracted" r-axis when imaging with 515 nm light, which must be multiplied by κ in order to reproduce the effectively "stretched" r-dimension when imaging with 700 nm light. Therefore, to transform the measured 515 nm heat distribution to what would have been measured with 700 nm light requires dividing the measured 515 nm data everywhere by κ^2 and stretching the radial position axis by a factor of κ.

Because the heat distribution expands over time, the correction factor κ is time-dependent:

$$\kappa(t) = \frac{\sigma_{\mathrm{meas},700}(t)}{\sigma_{\mathrm{meas},515}(t)} = \frac{\sqrt{\sigma_{\mathrm{pop}}^2(t) + \sigma_{700}^2}}{\sqrt{\sigma_{\mathrm{pop}}^2(t) + \sigma_{515}^2}} \tag{12}$$

so that every time point must be amplitude- and width-corrected by a different (diminishing) factor (Figure S4a). $\kappa(t)$ is calculated by: (1) extracting $\sigma_{\mathrm{meas},515}(t)$ from the Gaussian fit to the raw data at each time delay, (2) calculating $\sigma_{\mathrm{pop}}(t)$ using (1) and Equation 6, and (3) calculating $\sigma_{\mathrm{meas},700}(t)$ using the result of (2) in Equation 8. The time-dependent correction

factors applied to the raw azimuthally averaged 515 nm data before subtracting from the raw azimuthally averaged 700 nm data are listed in Table S1.

Table S1: Time-dependent PSF correction values

time (ns)	κ
0.0	1.078
0.1	1.071
0.2	1.065
0.3	1.060
0.5	1.052
0.7	1.045
1.0	1.038
1.5	1.031
2.0	1.026
4.0	1.015
7.0	1.010

Without accounting for the finite size of the imaged excited population, one may be tempted to use the ratio of the resolution limits at the two imaging wavelengths ($700/515 \approx 1.4$) as a global correction factor. However, the more complete picture described here demonstrates the importance of including the generated population's finite width and its time evolution in our analysis (Figure S4a). We confirm that the time-dependent correction factor preserves the original measured dynamical information, including the mean squared expansion of the heat distribution, which should be invariant to imaging wavelength under identical excitation conditions (Figure S4b).

The PSF-corrected radial profile for the 515 nm (2.4 eV, green circles) signal at time zero is shown in Figure S5 for comparison to the raw 700 nm (1.8 eV, red) signal and extracted exciton profiles (purple) generated from scaled subtraction of the PSF-corrected and η-scaled 515 nm data from the 700 nm data (green stars representing heat at 1.8 eV). The width of the corrected 515 nm signal is still much narrower than the full extent of the 700 nm signal, indicating that different imaging PSFs at the two probe wavelengths are not sufficient to explain the discrepancy. Instead, the population giving rise to the 515 nm signal diffuses more slowly during the instrument response time and must therefore be a different energy population than what gives rise to the faster-diffusing bright signal at 700 nm.

This experimental observation is corroborated by a calculation that compares the relative strength of the excitonic contribution to the optical response based on the relative proximity of the probe energy to its nearest electronic resonance. The dispersive lineshapes in the transient response follow a $1/(\omega - \omega_0)$ scaling detuned from resonant frequency ω_0. The nearest electronic resonance to the 1.77 eV probe is at 1.79 eV (A exciton resonance) whereas the nearest electronic resonance to the 2.41 eV probe is at 2.07 eV (B exciton resonance). Furthermore, we estimate from the steady state photoluminescence spectrum in Figure 1c that the oscillator strength of the B exciton resonance is $10\times$ smaller than that of the A exciton resonance. Therefore, the relative excitonic contribution to the far-from resonant measurement is suppressed by $\frac{1/(2.41-1.79)+0.1/(2.41-2.07)}{1/(1.79-1.77)+0.1/(2.07-1.77)} \approx 0.038$, or a factor of 25. We deduce

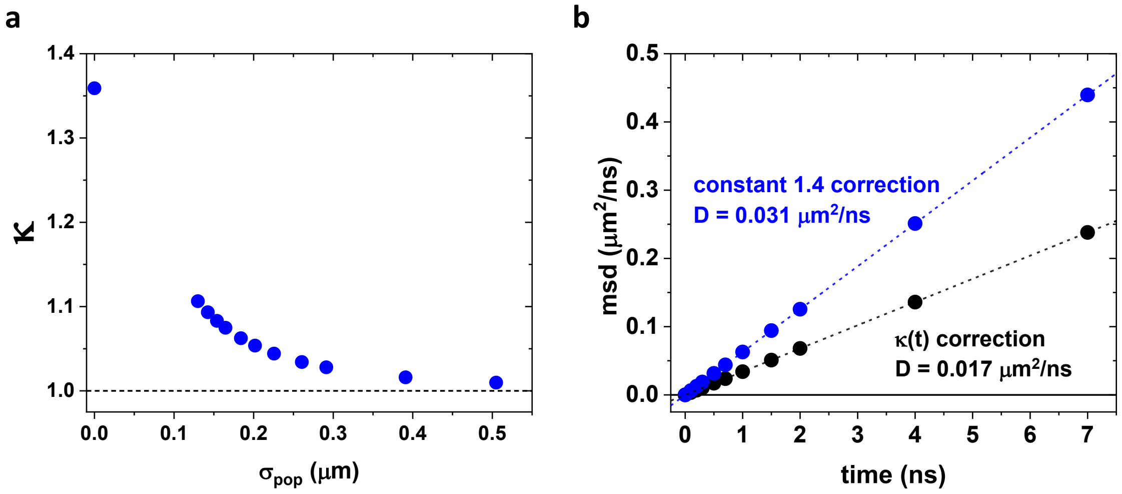

Figure S4: PSF correction. (a) Correction factor, κ, as a function of the actual population width, σ_{pop}. The maximum correction factor occurs for a delta function population and asymptotes to 1 (no correction) for increasingly broader populations, as expected. (b) Data transformation with a time-dependent correction factor $\kappa(t)$ (black) preserves the dynamics of the raw 515 nm data whereas a constant correction (blue) factor inflates the extracted diffusivity.

that this negligible excitonic contribution is overwhelmed by the heat-induced signal probed at 2.41 eV.

5. Temperature-dependent reflectance spectroscopy and construction of differential contrast due solely to heating

To identify the electronic resonances in our sample, we implement reflectance contrast spectroscopy. The same hBN-encapsulated 4L MoS_2 sample that was measured with stroboSCAT was also measured in a separate commercial inverted microscope adapted for reflectance contrast spectroscopy. The use of an air objective in the reflectance contrast microscope adds an additional index-mismatched interface that enhances the overall reflection compared to what would be measured with an oil immersion objective. We use transfer matrix simulations, described in the above section, to estimate a correction factor to convert between the air and oil immersion objective cases: $R_{\text{oil}} \backsimeq R_{\text{air}} - \frac{R_{0,\text{air}}}{2}$ where R is the reflectance under the sample and substrate and R_0 is the reflectance under the substrate only. The reflectance contrast, $RC = R/R_0$, may then be converted as $RC_{\text{oil}} = 2RC_{\text{air}} - 1$.

The lowest controlled heater set point (25°C) was used as a proxy for room temperature to normalize higher temperature set points in the construction of a differential reflectance spectrum due to sample heating:

$$\Delta R/R_{\text{heat}} = \frac{RC_{\text{hot}} - RC_{25^\circ\text{C}}}{RC_{25^\circ\text{C}}}, \tag{13}$$

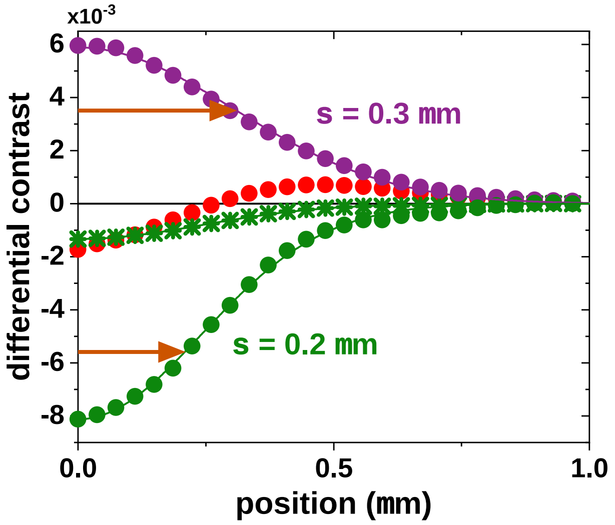

Figure S5: Time zero radial profiles of the 2.4 eV probe heat-dominated signal (green stars) following PSF correction, 1.8 eV probe raw signal (red), exciton profile (purple), and 1.8 eV heat profile (green circles).

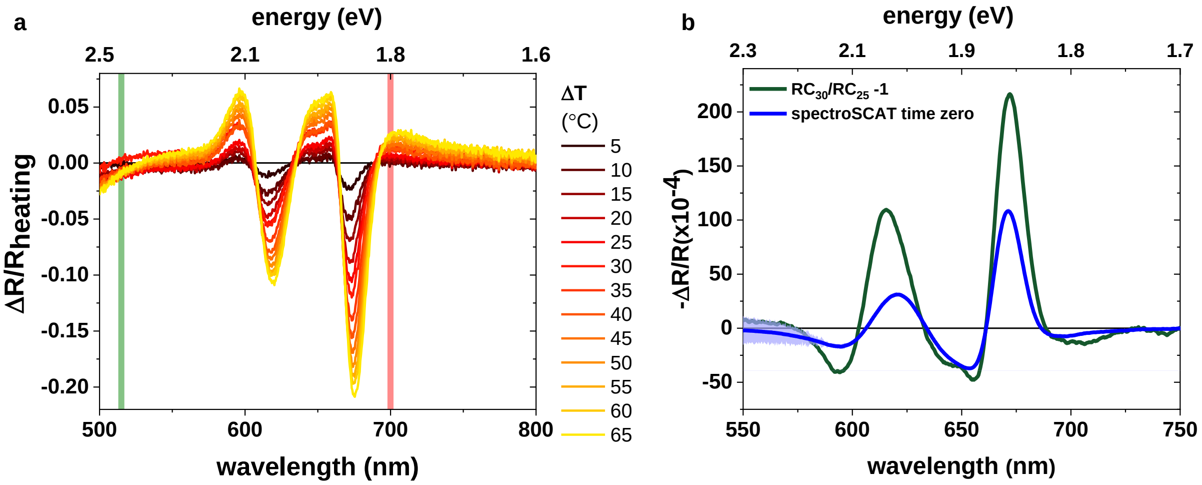

Figure S6: Reflectance spectroscopy as a function of temperature and compared to pump-induced differential reflectance (spectroSCAT). (a) Constructed differential reflectance spectra due to steady state heating normalized to the lowest controlled temperature set point spectrum (25°C). A zero-crossing near 2.4 eV (green line) further complicates reliable quantification of the optical response due to heating at this energy while the trend near 1.8 eV (red line) is clear and monotonic. (b) Expected differential contrast due to steady state heating by $\Delta T = 5$ K (green) and time-zero photoexcited differential reflectance spectra (blue) averaged over a ~500 nm line cut through the central excitation. Measurements are taken on the same 4L MoS_2 sample in separate instruments in the Atwater and Ginsberg groups, respectively. Shaded error bars on the spectroSCAT curve are estimated from the fluctuations of the white light probe pulse near the second harmonic.

yielding a qualitative representation of the optical material response due solely to heating (Figure S6a). Poor lamp spectral power below 550 nm adds significantly to the error near the zero crossing and therefore precludes quantitative analysis of the expected $\Delta R/R$ contrast due to heating in this spectral regime.

We note that the shape and location of resonance features in these differential heating spectra are strikingly similar to transient differential reflectance spectra measured after photoexcitation, suggesting that even modest sample heating of a few Kelvin dominates the transient response (Figure S6b). We collect transient reflectance spectra by focusing a white light probe onto the sample in the stroboSCAT microscope and dispersing the reflected light through a home-built prism spectrometer. The white light is generated by focusing the fundamental output (1030 nm, 200 kHz) of a Light Conversion PHAROS ultrafast regeneratively amplified laser system into a 3 mm sapphire crystal. The excitation source is the same diode laser that was used for experiments in the main text. An external delay generator, triggered with the pulse output of the ultrafast laser, controls the electronic delays between pump and probe. White light fluctuations near the second harmonic of the fundamental add significant noise below ~575 nm.

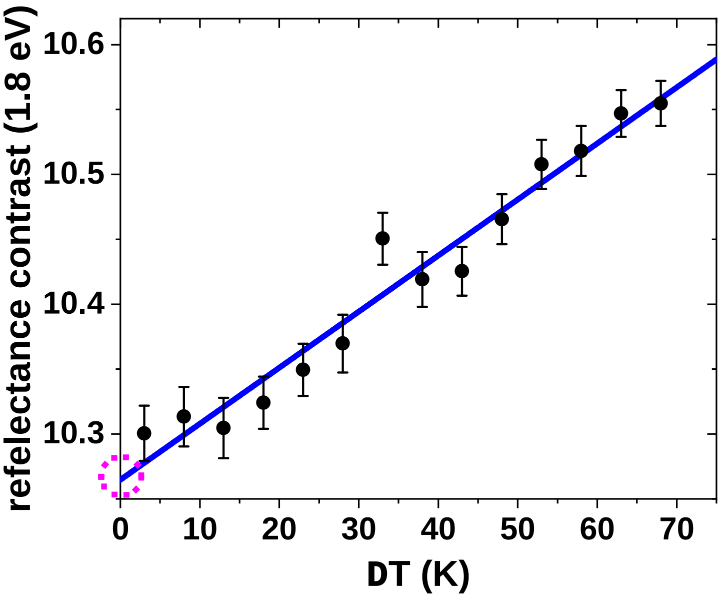

Figure S7: Reflectance contrast at 1.8 eV as a function of sample temperature elevation. The trend is extrapolated to room temperature ($\Delta T = 0$, pink dashed circle) using a linear fit (blue line). Error bars indicate the standard error of the mean propagated from averaging spectra and binning over the 4 nm laser line.

To estimate the temperature-dependent contribution to stroboSCAT contrast at the near-resonant 1.8 eV probe (Figure 2c in the main text), we first extrapolate the temperature-dependent reflectance contrast, binned over the 4 nm laser line, to room temperature (22°C in the temperature-controlled stroboSCAT laser lab) and use this value to normalize the expected differential contrast due to heating as in Equation 13 (Figure S7). Finally, for

direct comparison to stroboSCAT contrast, the result is multiplied by -1 to account for a relative difference of π in the Gouy focusing phase of the focused and widefield probes used in the reflectance contrast and stroboSCAT measurements, respectively.

6. Maximum sample temperature estimate from calorimetry

We perform a simple calorimetry calculation to estimate the maximum temperature in the sample if all absorbed photons were converted to heat. Using a peak carrier density in the center of the excitation spot of $N_{\text{peak}} = 3.5 \times 10^{13}$ cm^{-2}, pump energy of $E_{\text{pump}} = 2.8$ eV and a heat capacity[S6] of $c = 15.22$ cal/(mol·K) $= 3.27 \times 10^{12}$ eV·cm^{-2}K^{-2}, the maximum sample temperature increase is given by $\Delta T_{\text{max}} = N_{\text{peak}} E_{\text{pump}} c = 30$ K. Roughly half of this heat is due to thermalization to the indirect bandgap: $\Delta T_{\text{therm}} = N_{\text{peak}}(E_{\text{pump}} - 1.4 \text{ eV})c = 15$ K.

7. Long-time temperature decay scaling

The thermal conductivity of hBN is 15× greater than in MoS_2, therefore we assume the heat transfer to hBN to be unidirectional and irreversible. Initially, rapid interfacial transfer from MoS_2 to hBN occurs (few hundred ps), but once the temperature gradient has lessened, driving additional interfacial transfer is dependent on the time scale over which heat spreads throughout the hBN. If the very center ($r = 0$) of the original exciton profile (and resulting) temperature profile in MoS_2 is the most critical, we ask: How does the temperature of the hBN at $r = 0$ scale as a function of time? To answer this question, we assume that (1) initially the temperature profile in hBN mimics that of the MoS_2, i.e., is Gaussian, and that (2) the temperature profile in hBN evolves according to the heat equation, i.e., standard diffusion with a mean squared expansion governed by $\sigma^2(t) - \sigma^2(0) \sim D_{\text{hBN}} t$. The amplitude of a two-dimensional Gaussian profile that expands due to diffusion is given by

$$g(r = 0) = \frac{1}{\sigma\sqrt{2\pi}} \propto t^{-1/2}. \quad (14)$$

Therefore, once initial heat transfer is limited by the rate at which heat diffuses in the hBN, the rate at which the temperature lessens in the hBN will also determine the rate of interfacial transfer and scale as $t^{-1/2}$. We could not fit the heat profiles without the introduction of this $t^{-1/2}$ decay.

We note that this sensitivity to the kinetic scaling of heat transfer tracks the full thermal population decay, enabling stroboSCAT to measure the efficiency of interfacial energy transfer between different contact materials, e.g., between the light-absorbing layer and an intervening layer on the glass substrate.[S7]

8. Spatiotemporal model

Equations 1 and 2 in the main text are recast in natural units and expressed in matrix form for the `pdepe` function in MATLAB:

$$\frac{\partial}{\partial t}\begin{bmatrix}u_1\\u_2\end{bmatrix} = \frac{1}{r}\frac{\partial}{\partial r}\left(r\begin{bmatrix}\mathrm{A}u_2\partial_r u_1 + \mathrm{B}u_1\partial_r u_2\\ \partial_r u_2\end{bmatrix}\right) + \begin{bmatrix}-\frac{\tau_T}{\tau_X}u_1 - \tau_T R_{\text{A-M}}N_0 u_1^2 + \tau_T g - (\mathrm{A}+\mathrm{B})\partial_r u_1 \partial_r u_2\\ \alpha N_0 \mathrm{C} u_1^2 - (u_2 - 1) + \beta \mathrm{C} u_1 + \gamma \mathrm{C} g\end{bmatrix} \tag{15}$$

where

$$t' \equiv t/\tau_T \tag{16}$$

$$r' \equiv r/\sqrt{D_T \tau_T} \tag{17}$$

$$u_1(r', t') \equiv N(r,t)/N_0 \tag{18}$$

$$u_2(r', t') \equiv T(r,t)/T_0 \tag{19}$$

$$g(r', t') \equiv G(r,t)/N_0 \tag{20}$$

and we define

$$\mathrm{A} \equiv \frac{\mu k_B T_0}{q D_T} \tag{21}$$

$$\mathrm{B} \equiv \frac{q\mu s T_0}{D_T} \tag{22}$$

$$\mathrm{C} \equiv \frac{N_0 \tau_T}{T_0} \tag{23}$$

The initial conditions are set to be $N(r,0) = 0$ and $T(r,0) = 300$ K. The boundary conditions are set so that the exciton and temperature fluxes go to zero.

To model the pump pulse, which has a finite duration, we use a generating function, $G(r,t)$, which is a product of two Gaussian functions. The first is a Gaussian in space with a standard deviation of σ_r. At the center, the peak exciton density is N_0. The second is a Gaussian in time with a standard deviation of σ_t and normalized to 1. Therefore,

$$G(r,t) = N_0 \exp\left[\frac{-r^2}{2\sigma_r^2}\right]\frac{1}{\sqrt{2\pi\sigma_t^2}}\exp\left[\frac{-(t-t_0)^2}{\sigma_t^2}\right] \tag{24}$$

or in natural units

$$g(r', t') = \frac{1}{\tau_T\sqrt{2\pi\sigma_t^2}}\exp\left[\frac{-r^2}{2\sigma_r^2}\right]\exp\left[\frac{-(t-t_0)^2}{\sigma_t^2}\right] \tag{25}$$

The exciton mobility and lifetime are allowed to vary over the range $15 < \mu < 22$ cm^2/V·s

and $1 < \tau_X < 23$ ns, constrained by the experimentally extracted diffusivity and long decay time constants across exciton profiles extracted from $1.4 < \eta < 7$. The A-M coefficient, $R_{\text{A-M}}$, is constrained from estimated literature values to $5 \times 10^{-5} < R_{\text{A-M}} < 5 \times 10^{-2}$ cm^2/s. The heat diffusivity and lifetime are fixed by the experimentally measured values. We estimate that, typical for few-layer MoS_2 where light emission from the indirect band gap must be phonon-assisted, the photoluminescence quantum yield (PLQY) is ∼1%, although we found that the peak time-zero temperature is insensitive to the value of the PLQY used.

We used this spatiotemporal model to self-consistently determine η,

$$\eta = \frac{-0.00042(5)/\text{K} \times \Delta T_{\text{max}}}{-0.00124(2)} \tag{26}$$

using the following process. We extracted exciton profiles for a given value of η, then ran the spatiotemporal model optimization on the experimental exciton and heat data. If the η value predicted from the best fit maximum time-zero temperature, $\Delta T_{\text{max,fit}}$, did not agree with the value of η used to generate the exciton profiles for the fit, then we extracted a new set of exciton profiles using the η value predicted by $\Delta T_{\text{max,fit}}$ in Equation 26 and performed the optimization again until the η value predicted by $\Delta T_{\text{max,fit}}$ in Equation 26 and the η value used for the exciton profiles in the optimization agreed.

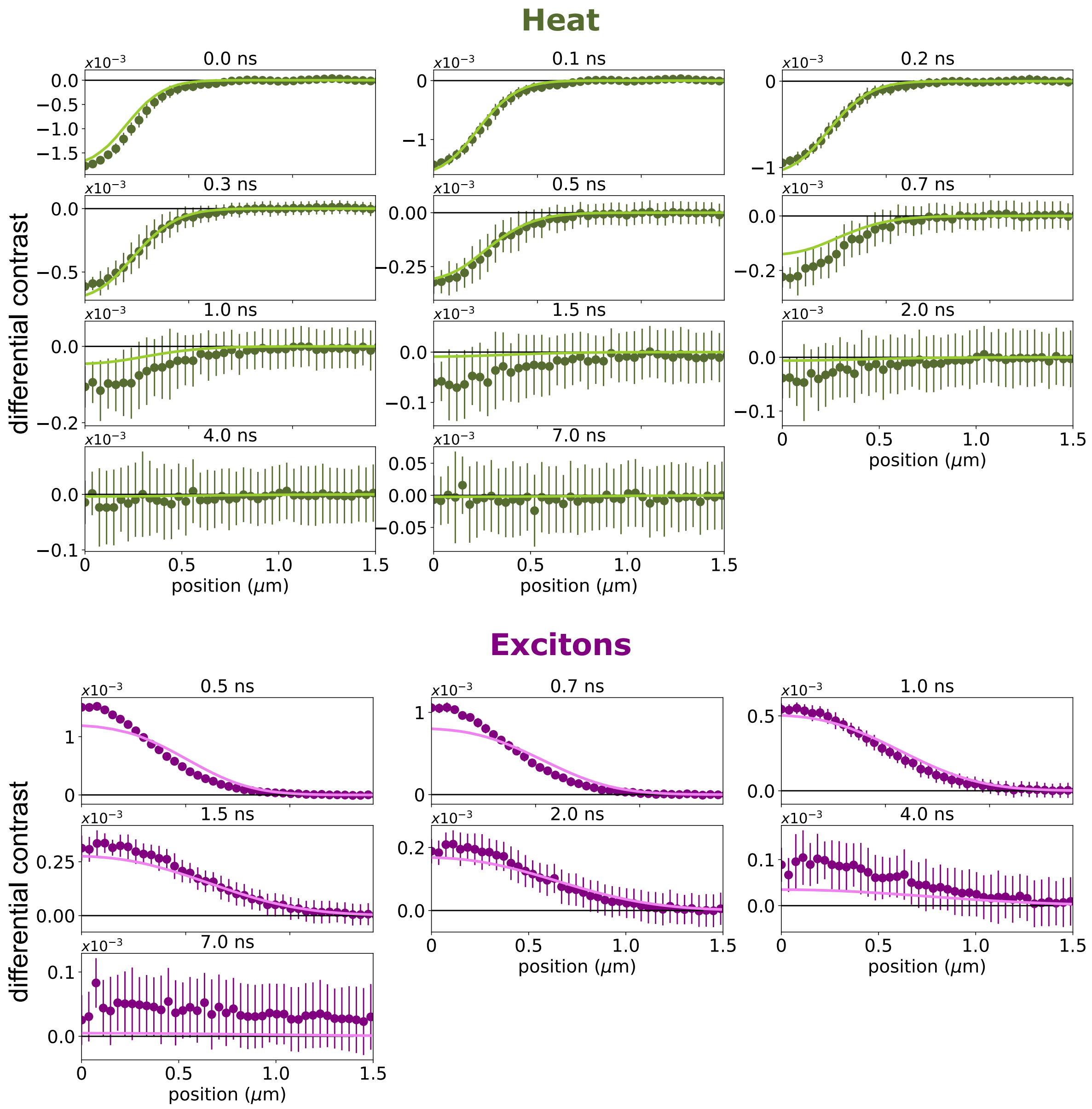

Figure S8: (top) Experimental far-from resonant heat profiles (dark green) with best fit from spatiotemporal model (light green). (bottom) Isolated experimental exciton profiles (dark purple) with best fit from spatiotemporal model (light purple).

9. Spatiotemporal kinetic model with Perea-Causín et al. experimental parameters

Below are all model parameter values employed in two separate simulations. The right column summarizes the values either constrained or obtained via (experimentally bounded) fitting to the experimental stroboSCAT data. The middle column lists the corresponding

parameters taken from Perea Causín et al.[S8] to reflect that the model generates "halo" profiles consistent with their experimental observations using microTRPL.

Table S2: Spatiotemporal model parameters

	Perea-Causín et al.[S8]	This work
τ_{X} [ns]	0.7	4.1
D_{X} [cm^2/s]	0.3	0.57
$R_{\text{A-M}}$ [cm^2/s]	0.5	0.00026
σ_{pump} [nm]	174	168
t_{pump} [ps]	0.1	72
E_{pump} [eV]	2.43	2.82
E_{BG} [eV]	2.05	1.38
N_{peak} [cm^{-2}]	7×10^{12}	3.5×10^{13}
c [J/g·K]	0.3	0.4
τ_{T} [ns]	0.4	0.3
D_{T} [cm^2/s]	0.05	0.2
PLQY [%]	1	1
S [µV/K]	1,000	0

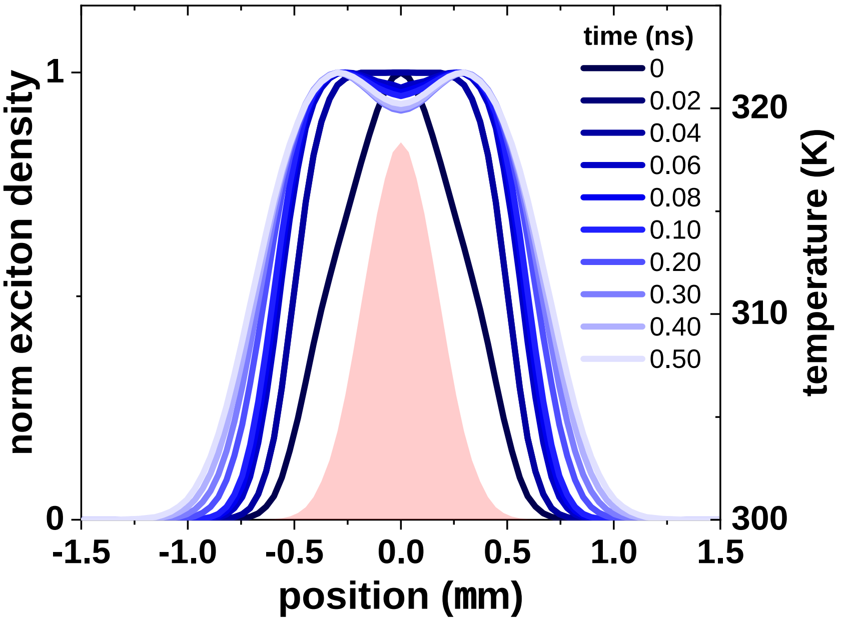

Figure S9: Normalized exciton profiles (blue) and time-zero temperature gradient (pink shaded curve) predicted with a spatiotemporal kinetic model that includes a Seebeck driving term in the exciton evolution equation over the same time delays measured in Figure 4 of Perea-Causín et al.[S8]

References

(S1) Byrnes, S. J. Multilayer optical calculations. 2020; `http://arxiv.org/abs/1603.02720`.

(S2) Byrnes, S. tmm: Simulate light propagation in multilayer thin and/or thick films using the fresnel equations and transfer matrix method. `http://pypi.python.org/pypi/tmm`.

(S3) Song, B.; Gu, H.; Fang, M.; Chen, X.; Jiang, H.; Wang, R.; Zhai, T.; Ho, Y.-T.; Liu, S. Layer-Dependent Dielectric Function of Wafer-Scale 2D MoS2. *Advanced Optical Materials* **2019**, *7*, 1801250.

(S4) Lee, S.-Y.; Jeong, T.-Y.; Jung, S.; Yee, K.-J. Refractive Index Dispersion of Hexagonal Boron Nitride in the Visible and Near-Infrared. *physica status solidi (b)* **2019**, *256*, 1800417.

(S5) Li, X.; Zhu, H. Two-dimensional MoS2: Properties, preparation, and applications. *Journal of Materiomics* **2015**, *1*, 33–44.

(S6) Volovik, L. S.; Fesenko, V. V.; Bolgar, A. S.; Drozdova, S. V.; Klochkov, L. A.; Primachenko, V. F. Enthalpy and heat capacity of molybdenum disulfide. *Soviet Powder Metallurgy and Metal Ceramics* **1978**, *17*, 697–702.

(S7) Utterback, J. K.; Sood, A.; Coropceanu, I.; Guzelturk, B.; Talapin, D. V.; Lindenberg, A. M.; Ginsberg, N. S. Nanoscale Disorder Generates Subdiffusive Heat Transport in Self-Assembled Nanocrystal Films. *Nano Letters* **2021**, *21*, 3540–3547.

(S8) Perea-Causín, R.; Brem, S.; Rosati, R.; Jago, R.; Kulig, M.; Ziegler, J. D.; Zipfel, J.; Chernikov, A.; Malic, E. Exciton Propagation and Halo Formation in Two-Dimensional Materials. *Nano Letters* **2019**, *19*, 7317–7323.
